# Thermal Preference Ranges Correlate with Stable Signals of Universal Stress Markers in Lake Baikal Endemic and Holarctic Amphipods

**DOI:** 10.1371/journal.pone.0164226

**Published:** 2016-10-05

**Authors:** Denis Axenov-Gribanov, Daria Bedulina, Zhanna Shatilina, Lena Jakob, Kseniya Vereshchagina, Yulia Lubyaga, Anton Gurkov, Ekaterina Shchapova, Till Luckenbach, Magnus Lucassen, Franz Josef Sartoris, Hans-Otto Pörtner, Maxim Timofeyev

**Affiliations:** 1 Institute of Biology at Irkutsk State University, Irkutsk, Russia; 2 Alfred Wegener Institute Helmholtz Centre for Polar and Marine Research, Bremerhaven, Germany; 3 Helmholtz Centre for Environmental Research–UFZ, Leipzig, Germany; 4 University of Bremen, Bremen, Germany; 5 Baikal Research Centre, Irkutsk, Russia; University of Arkansas for Medical Sciences College of Pharmacy, UNITED STATES

## Abstract

Temperature is the most pervasive abiotic environmental factor for aquatic organisms. Fluctuations in temperature range lead to changes in metabolic performance. Here, we aimed to identify whether surpassing the thermal preference zones is correlated with shifts in universal cellular stress markers of protein integrity, responses to oxidative stress and lactate content, as indicators of anaerobic metabolism. Exposure of the Lake Baikal endemic amphipod species *Eulimnogammarus verrucosus* (Gerstfeldt, 1858), *Ommatogammarus flavus* (Dybowski, 1874) and of the Holarctic amphipod *Gammarus lacustris* Sars 1863 (Amphipoda, Crustacea) to increasing temperatures resulted in elevated heat shock protein 70 (Hsp70) and lactate content, elevated antioxidant enzyme activities (i.e., catalase and peroxidase), and reduced lactate dehydrogenase and glutathione S-transferase activities. Thus, the zone of stability (absence of any significant changes) of the studied molecular and biochemical markers correlated with the behaviorally preferred temperatures. We conclude that the thermal behavioral responses of the studied amphipods are directly related to metabolic processes at the cellular level. Thus, the determined thermal ranges may possibly correspond to the thermal optima. This relationship between species-specific behavioral reactions and stress response metabolism may have significant ecological consequences that result in a thermal zone-specific distribution (i.e., depths, feed spectrum, etc.) of species. As a consequence, by separating species with different temperature preferences, interspecific competition is reduced, which, in turn, increases a species’ Darwinian fitness in its environment.

## Introduction

Temperature is the most pervasive abiotic environmental factor for aquatic ectotherms. Growth, development, ontogenesis and metabolism of organisms depend on the thermal regime. The conformation and activity of macromolecules, metabolic rate and energetic status of organisms are affected by temperature. Thus, a change in ambient temperature leads to a shift in the metabolic regulation system [1–3].

Stenothermal species are narrowly adapted to thermal habitats where their metabolic processes function with high efficiency. These patterns contrast with observations of eurythermal organisms, which are adapted to comparatively wide thermal ranges. Fluctuations in environmental parameters, such as temperature, lead to changes in metabolic performance [4]. Thermal stress reactions in organisms emerge because of temperature fluctuations that go beyond optimal conditions. In addition, most aquatic organisms, which are able to actively migrate within their habitat, have developed behavioral responses to temperature changes that allow maintenance of highly efficient functioning of physiological processes [5–7].

There are several approaches used to define the thermal tolerance of living organisms and to predict the ecosystem reaction to climate change. The most common of these approaches is the exposure of experimental animals to a range of temperatures while investigating the critical (CTmax) or lethal temperature (LT) for each species [8, 9]. In nature, however, this approach has certain limitations. For instance, a species might acclimate to certain temperatures that are critical in short-term experiments or it may migrate to avoid deleterious temperatures. Therefore, behavioral experiments are needed to gain a comprehensive picture of species-specific responses to temperature. However, the relationship between thermal preference behaviors and universal cellular stress response is not entirely understood. Moreover, the link between the optimum temperature for metabolic processes and thermal preference behavior in a natural habitat of a species still needs to be investigated. From an experimental perspective, organisms that have evolved under stable and constant environmental conditions are the best candidates to study because deviations from their natural habitat temperatures can be distinctively mimicked.

The endemic fauna of Lake Baikal, the world’s oldest (25–30 million years) freshwater lake, offers great preconditions for such a study, as it has been characterized by stable abiotic and biotic conditions, which have prevailed for millions of years. These conditions include low mineralization and organic content, high oxygen content (12 mg/L) and stable low temperatures (3.5 to 6°C at depths of 30–100 m and a constant temperature of 3.5°C at depths below 100 m), which probably promote the unique, high level of biodiversity (more than 2600 species) and endemism (approximately 80%) [10, 11]. Amphipods (Amphipoda, Crustacea) are one of the largest groups of macroinvertebrates, represented by more than 355 species (degree of endemism: 100%) in Lake Baikal [12, 13]. They are key benthic decomposers in Lake Baikal as in most other freshwater and marine environments.

In contrast to the Lake Baikal ecosystem, ecosystems of shallow water reservoirs of the Holarctic are characterized by various levels of mineralization, organic content, and thermal regimes and by a low concentration of dissolved oxygen (less than 7 mg/L).

By exposing amphipod species *O*. *flavus*, *E*. *verrucosus* and *G*. *lacustris*, which differ in thermal resistance and preference, to increasing/decreasing ambient temperatures, we aimed to identify whether surpassing the thermal preference zones is correlated with shifts in universal cellular stress markers of protein integrity, responses to oxidative stress and lactate content as indicators of anaerobic metabolism.

## Materials and Methods

### Experimental animals

Animals were collected lake Baikal close to Bolshie Koty settlement (Irkutsk region, Eastern Siberia, Russia). No specific permissions were required for these sampling. No specific permissions were required for these locations and activities. Amphipods species not involve endangered or protected species.

Two endemic amphipod species, *Eulimnogammarus verrucosus* (Gerstfeldt, 1858) and *Ommatogammarus flavus* (Dybowski, 1874), and the Holarctic amphipod *Gammarus lacustris* Sars, 1863 were chosen as experimental species for this study.

*E*. *verrucosus* is omnivorous and typically inhabits the Lake Baikal littoral at depths ranging from 0 to 15 meters. This species is cryophilic (thermal preference zone 3°C– 8°C [14], stenothermic and reproduces in the winter [15]. It was assumed that *E*. *verrucosus* migrates to deeper benthic zones during the summer due to increasing temperatures in the littoral zone [16].

*O*. *flavus* is a cryophilic (thermal preference zone 3°C– 8°C [14], stenothermic benthic scavenger and a major component of Lake Baikal’s deep water fauna. This species is eurybathic and is found at depths between 2.5 and 1313 m. The preferred depth is greater than 100 meters [17]. As this species tolerates a wide range of water pressures, it can be acclimated to laboratory conditions without loss of activity or feeding rate [18].

*G*. *lacustris* is a representative of the eurythermic Holarctic fauna and typically inhabits stagnant water bodies with fluctuating abiotic conditions, such as varying temperatures (from 0 up to 30°C) or changing salinities and oxygen content [19, 20]. Multiple reproduction periods may occur depending on the environmental and ecological characteristics of the habitat. In the Baikal region, this species inhabits shallow lakes and ponds in the vicinity of Lake Baikal [21, 22]. *G*. *lacustris* can be found in some shallow water bays (named sors) of Lake Baikal [23]. The abiotic parameters of these sors are close to Holarctic water reservoirs. However, *G*. *lacustris* is not found in waters of the open Lake Baikal.

It is known that the thermal tolerance of studied species reflects *O*. *flavus* < *E*. *verrucosus* < *G*. *lacustris* [14]. Thus, the thermal tolerance of Holarctic amphipods *G*. *lacustris* is much higher than thermal tolerance of *E*. *verrucosus* inhabiting the Baikal littoral. The thermal tolerance of *O*. *flavus*, which inhabits deeper waters of Baikal, is lower than that of *E*. *verrucosus*. The characterization of thermal preference behavior of the amphipod species were obtained by using a thermal gradient chamber. This chamber allows animals to select their preferred thermal zone in gradual thermal range of 3 to 22°C and it is presented in [24, 25] and S1 Table.

### Sampling and laboratory acclimation

Adult individuals of *E*. *verrucosus* (body length 33 ± 3 mm, mean +/- stdev, n = 200) were collected using a hand net near the village of Listvyanka (South Baikal N 51.852083, E 104.865268) in the winter and spring of 2009 – 2010 at temperatures of 4–5°C. Adult *O*. *flavus* specimens (body length 19 ± 2 mm) were collected using baited benthic traps with rotten fish at depths of 50–100 meters near Bolshie Koty (South Baikal N 51.903375, E 105.075639) in the winter and spring of 2011. The temperature at these depth zones of Lake Baikal is 3.5–3.6°C [12]. Adult *G*. *lacustris* individuals (body length 18 ± 2 mm, mean +/- stdev, n = 250) were collected using a hand net in the spring of 2010 in a backwater of the Angara river (N 52.26798, E 104.28127) located in the city of Irkutsk at the temperature of 5–6°C.

Immediately after sampling, the amphipods were transferred to 2 L containers filled with constantly aerated Baikal water at water temperatures of 6°C (*E*. *verrucosus*, *G*. *lacustris*) and 4°C (*O*. *flavus*). The sampling temperature was 5°C for *E*. *verrucosus* and *G*. *lacustris* and 3.5°C for *O*. *flavus*. The amphipods were transported to the laboratory at Irkutsk State University where the animals were acclimated to the laboratory conditions for 4 – 7 days at the same temperatures as sampling and transportation. The conditions for pre-acclimation corresponded to the annual average temperatures of the habitats of the respective species. During the acclimation period, the amphipods were fed *ad libitum* with Tetramin (Tetra, Melle, Germany). No mortality of the amphipods was observed during acclimation to laboratory conditions.

### Experimental design

For investigating the cellular stress response and mortality rate, two types of experiments were carried out. In the first experiment (modified after Sokolova and Pörtner [26]) amphipods were exposed to gradually increasing temperatures (1°C h^-1^), starting from a pre-acclimation (6°C for *E*. *verrucosus* and *G*. *lacustris*, and 4°C for *O*. *flavus*) until reaching a temperature at which the cumulative mortality was 50%. Second, (modified after Sokolova and Pörtner [26]) the amphipods were exposed to gradually decreasing temperatures at the same rate of change as during the warming protocols, again starting from the pre-acclimation temperature and continuing until reaching 0.5°C (close to freezing).

Upon each temperature increase step of 2°C (i.e., every 2 h) and each decrease step by 1°C (i.e., every 1 h), several specimens (2 for *E*. *verrucosus*, 3 for *O*. *flavus* and 5 for *G*. *lacustris*) were collected from each of the well-aerated water tanks (n = 3–7) and shock-frozen in liquid nitrogen. Control samples were collected from water tanks kept at the respective initial acclimation temperatures (see above).

All experiments were carried out using a circulation thermostat WiseCircu WCR-P8 (Daihan Scientific, Korea). The temperature was increased manually every 10 minutes to provide an even speed of temperature increase by 1°C per hour, and the temperature was recorded. Temperature control was performed directly in each aquarium using digital thermometers. Prior to exposure, test runs of the temperature increase and decrease were performed. The durations of gradual increasing and decreasing experiments were 24 h and 6 h, respectively. Approx. 1000 individuals of *E*. *verrucosus*, 850 individuals of *O*. *flavus*, and 2600 individuals of *G*. *lacustris* were analyzed in total.

For the mortality experiments (n = 7–11 for each species), 10 specimens of *E*. *verrucosus* and *O*. *flavus* and 15 specimens of *G*. *lacustris* were exposed in well-aerated aquaria to gradually change the temperature (1°C h^-1^), starting from the pre-acclimation temperature (6°C for *E*. *verrucosus* and *G*. *lacustris*, and 4°C for *O*. *flavus)* and continuing to increase or decrease the water temperature until 100% mortality was observed. Dead animals were recorded every hour and removed from the experiments.

### Determination of universal cellular stress markers

Several universal cellular stress markers were investigated upon gradually increasing and decreasing the temperatures, including the level of the heat shock protein Hsp70, the activities of antioxidative enzymes (peroxidase, catalase and glutathione S-transferase), lactate content, and lactate dehydrogenase activity.

The level of Hsp70 was determined using SDS-PAGE followed by Western blotting, as described in detail by Axenov-Gribanov et al. [27] and Bedulina et al. [28]. The following Hsp70 antibodies were used: monoclonal anti-Hsp70, produced in mouse (# H5147, Sigma Chemical Co, dilution 1:5000) as a primary antibody and secondary polyclonal anti-mouse antibody (Stressgen, # SAB-101, dilution 1:1000). As a reference control, actin levels were determined: polyclonal anti-β-actin antibody produced in rabbit (# A2668, Sigma Chemical Co, dilution 1:1000) and secondary anti-rabbit antibody (# A9919, Sigma Chemical Co, dilution 1:1000). Protein-antibody complexes were detected using 5-bromo-4-chloro-3-indolyl phosphate disodium salt and nitrotetrazolium blue chloride (BCIP-nBT, # B6149 (Sigma), # N6876 (Sigma)). Hsp70 and actin levels were measured by semi-quantitative analysis of grey values on scanned Western blot membranes using ImageJ software with the Fiji package [29]. The levels of Hsp70 were normalized relative to β-actin expression in each sample.

The activities of antioxidative enzymes (peroxidase, catalase, glutathione S-transferase) were determined using the methods described by Drotar et al. [30], Aebi [31] and Habig et al. [32], respectively, and modified according to Timofeyev et al. [18] and Bedulina et al. [28]. Amphipods (2 specimens of *E*. *verrucosus* and 3 specimens of *O*. *flavus* and *G*. *lacustris* for each sample) were homogenized in 0.1 M sodium-phosphate buffer (pH 6.5) and centrifuged at 10 000 rpm (3 min at 4°C). Hydrogen peroxide, hydrogen peroxide with guaiacol (# G5502, Sigma) and 1-chloro-2,4-dinitrobenzene (# 237329, Sigma) were used as substrates for catalase, peroxidase and glutathione S-transferase, respectively. The spectrophotometric assays were performed at 25°C and at λ = 340 nm for peroxidase, λ = 240 nm for catalase, and λ = 436 nm for glutathione S-transferase.

Lactate levels were determined using a Lactate-vital express kit (Vital–Diagnostics, St. Petersburg, Russia) according to Bergmeyer [33]. Spectrophotometry was performed using a Cary 50 spectrophotometer (Varian, USA) at λ = 505 nm. The activity of lactate dehydrogenase was determined by an “LDH-vital” express kit (Vital–Diagnostics Spb, Russia) at λ = 340 nm. For measurements of lactate concentration and lactate dehydrogenase activity amphipods were pooled as same, as used in other assays (2 specimens of *E*. *verrucosus* and 3 specimens of *O*. *flavus* and *G*. *lacustris* for each sample)

### Statistics

All experiments were carried out in 3 – 7 biological replicates, and biochemical measurements were performed in triplicate for each sample. All data are presented in Supplementary materials (S1–S8 Tables). Control groups included animals that were exposed to temperatures corresponding to the annual temperatures of the environment (*O*. *flavus*: 4°C; *E*. *verrucosus*: 6°C and *G*. *lacustris*: 6°C). The Shapiro-Wilk test revealed that all data sets were normally distributed. The following parametric statistical tests were subsequently applied: The uniformity of variance was tested by the Flinger test before conducting a one-way analysis of variance (ANOVA using general linear model). Welch's method was applied when variances were not uniform. Differences were considered to be significant at p < 0.05 (alpha 0.05) (Table 1). After performing an ANOVA, all experimental groups were compared with their control groups using Dunnett's test. Spearman’s rank correlation analysis was performed for all studied species and all tested biochemical parameters.

**Table 1:** ANOVA: Effects of gradient temperature change on Hsp70 and lactate contents and on the activities of peroxidase, catalase, glutathione S-transferase and lactate dehydrogenase in *E*. *verrucosus*, *O*. *flavus*, and *G*. *lacustris*.

	*E*. *verrucosus*	*O*. *flavus*	*G*. *lacusrtis*
**Hsp70**	F_1.89_ = 4.15, P<0.001***	F_3.92_ = 7.31, P = 0.011**	F_1.86_ = 3.68, P<0.001***
**Lactate**	F_1.96_ = 20.48, P<0.001***	F_2.41_ = 11.53, P<0.001***	F_2_ = 19.29, P<0.001***
**Peroxidase**	F_1.74_ = 11.48, P<0.001***	F_2.12_ = 4.92, P<0.001***	F_1.7_ = 7.72, P<0.001***
**Catalase**	F_1.74_ = 19.11, P<0.001***	F_3.07_ = 3.77, P = 0.027*	F_1.69_ = 1.4, P = 0.145
**Glutathione S-transferase**	F_1.73_ = 6.87, P<0.001***	F_2.12_ = 3.79, P = 0.002**	F_1.69_ = 2, P = 0.014**
**Lactate dehydrogenase**	F_1.99_ = 28.52, P<0.001***	F_2.12_ = 11.07, P<0.001***	F_1.7_ = 10.81, P<0.001***

Asterisks in Table 1 indicates statistically significant differences from controls at p < 0.05(*), p < 0.01 (**), and p < 0.001 (***).

Statistical analysis was performed using the statistics software R (R Core Team, 2011) with additional *multcomp* and *sandwich* packages [34,35,36].

Mortality data were fitted to the Weibull model [37] in R. LT50 (the temperature at which mortality of 50% of individuals occurred) values were derived from the regressions using a following equation:

$$m=100-\frac{100}{e^{{\left(\frac{t}{p}\right)}^{r}}}$$ (1)

*m*–cumulative part of dead individuals, %

*t*–temperature, °C

*p* and *r*–regression coefficients

## Results

### Thermal preference behavior

**Fig 1:**
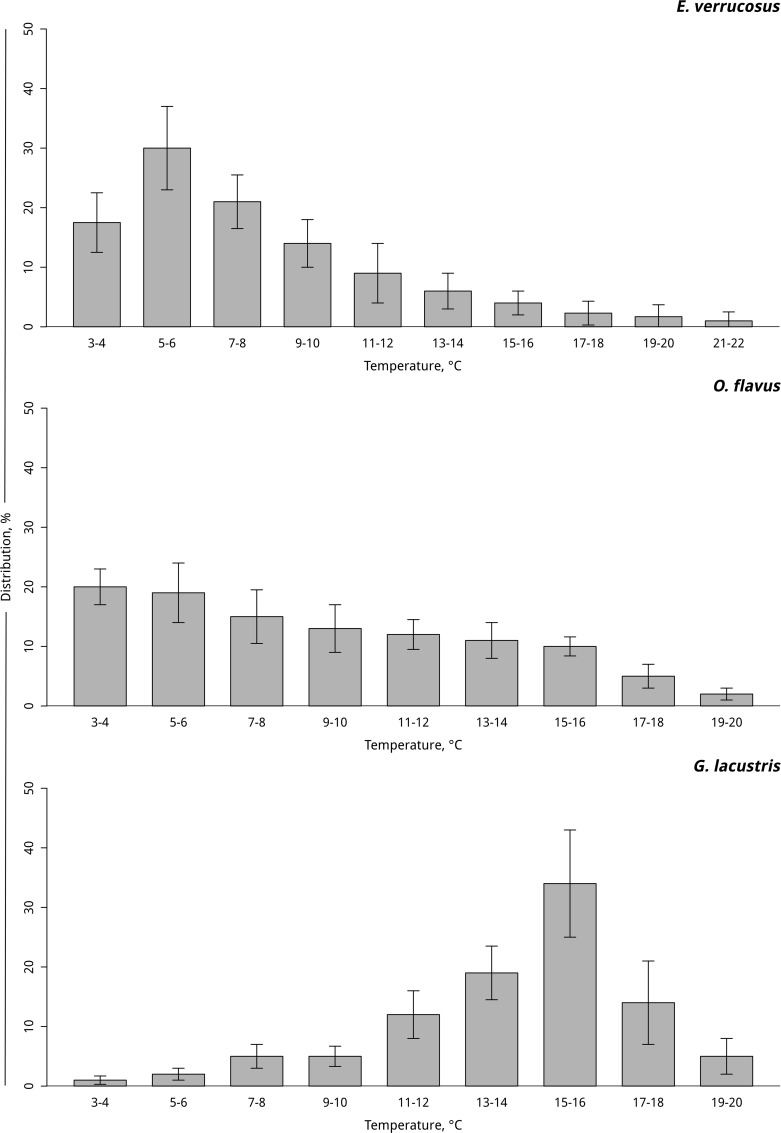
Distribution of *E*. *verrucosus* (n = 5), *O*. *flavus* (n = 4) and *G*. *lacustris* (n = 5) individuals in an experimental thermal gradient. Additional information presented in S1 Table.

A summary of previously published results [24, 25] is presented in Fig 1, and S1 Table shows the different degrees of thermal preference behavior. According to these experiments, *E*. *verrucosus* mainly selected a thermal zone of 3–10°C, with the highest percentage of animals (up to 30%) found in the temperature range of 5–6°C. Most individuals of *O*. *flavus* preferred temperatures of 3–8°C (up to 50%). In contrast to the Lake Baikal endemic species, the common Holarctic *G*. *lacustris* preferred significantly higher temperatures (i.e., 11–18°C) with most individuals choosing a thermal zone of 15–16°C (up to 40%).

### Thermal resistance

**Fig 2:**
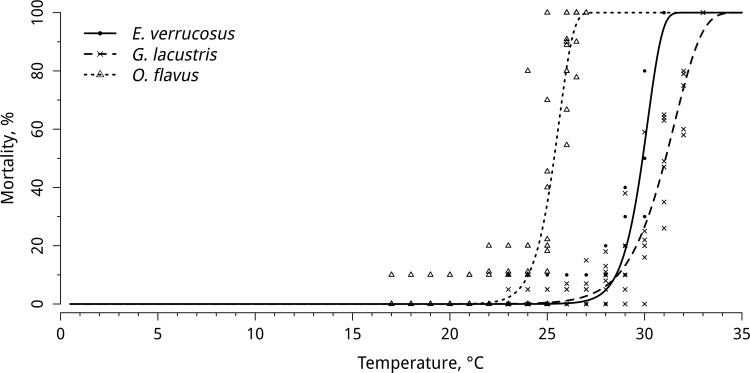
Mortality of *E*. *verrucosus* (n = 7), *O*. *flavus* (n = 11) and *G*. *lacustris* (n = 7) during exposure to a gradual temperature increase/decrease. The figure includes data on gradually increasing hypothermia and hyperthermia experiments. Additional information presented in S2 Table.

The mortalities of the studied amphipod species upon exposure to the experimental conditions are presented in Fig 2 and S2 Table. The LT50 values were 23°C for *O*. *flavus*, 29.5°C for *E*. *verrucosus*, and 31°C for *G*. *lacustris*. No mortality of any of the investigated species was observed during gradual temperature decrease; however, at temperatures below 2°C, specimens of *G*. *lacustris* fell into cold stupor, which was determined by the visible decrease in locomotion activity and the decrease of pleopods beating, while the animals remained alive, and activity could be restored when returned to warm water.

### Universal cellular stress markers

#### Hsp 70

**Fig 3:**
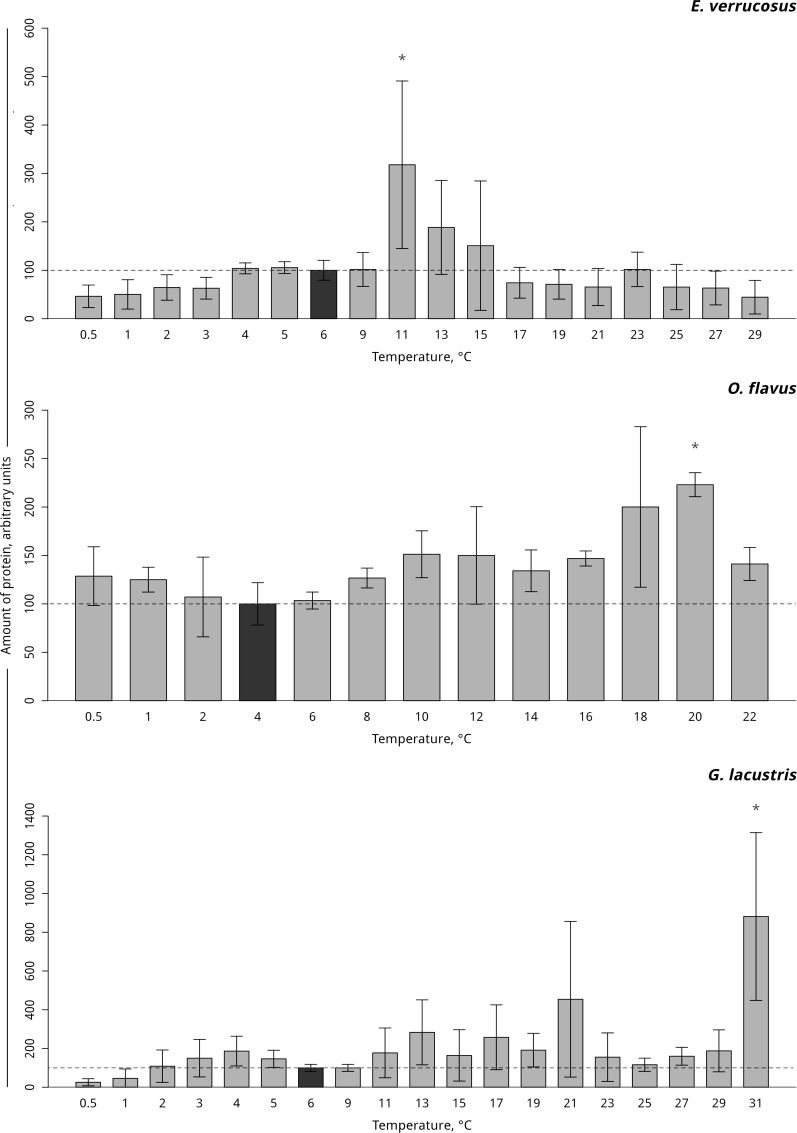
Hsp70 levels in amphipod species during exposure to gradually changing temperatures. **The figure includes data on gradually increasing hypothermia and hyperthermia experiments.** *Indicates a significant difference (p<0.05) from the control (*O*. *flavus*: 4°C; for *E*. *verrucosus*: 6°C and *G*. *lacustris*: 6°C). The dark grey columns and dotted line indicate the control level. Number of replicates: *O*. *flavus* (n_control_ = 4, n_exp._ = 4); *E*. *verrucosus* (n_control_ = 8, n_exp._ = 5) and *G*. *lacustris* (n_control_ = 5, n_exp._ = 5). Additional information presented in S3 Table.

Hsp70 was found to be constitutively expressed in controls without stress impact in all three species. In the thermosensitive littoral Baikal species *E*. *verrucosus*, gradual temperature increase led to a significant three-fold elevation in the level of Hsp70 at 11°C (p < 0.001) with a subsequent decline to the control level. In the thermosensitive deepwater Baikal species *O*. *flavus*, a significant two-fold increase in the level of Hsp70 occurred at 20°C (p < 0.001). A significant seven-fold increase in the level of Hsp70 in the thermotolerant Holarctic species *G*. *lacustris* was detected at 31°C (p < 0.001). Gradual temperature decrease did not alter the level of Hsp70 in all studied species (Fig 3, S3 Table).

#### Antioxidative enzymes activities

**Fig 4:**
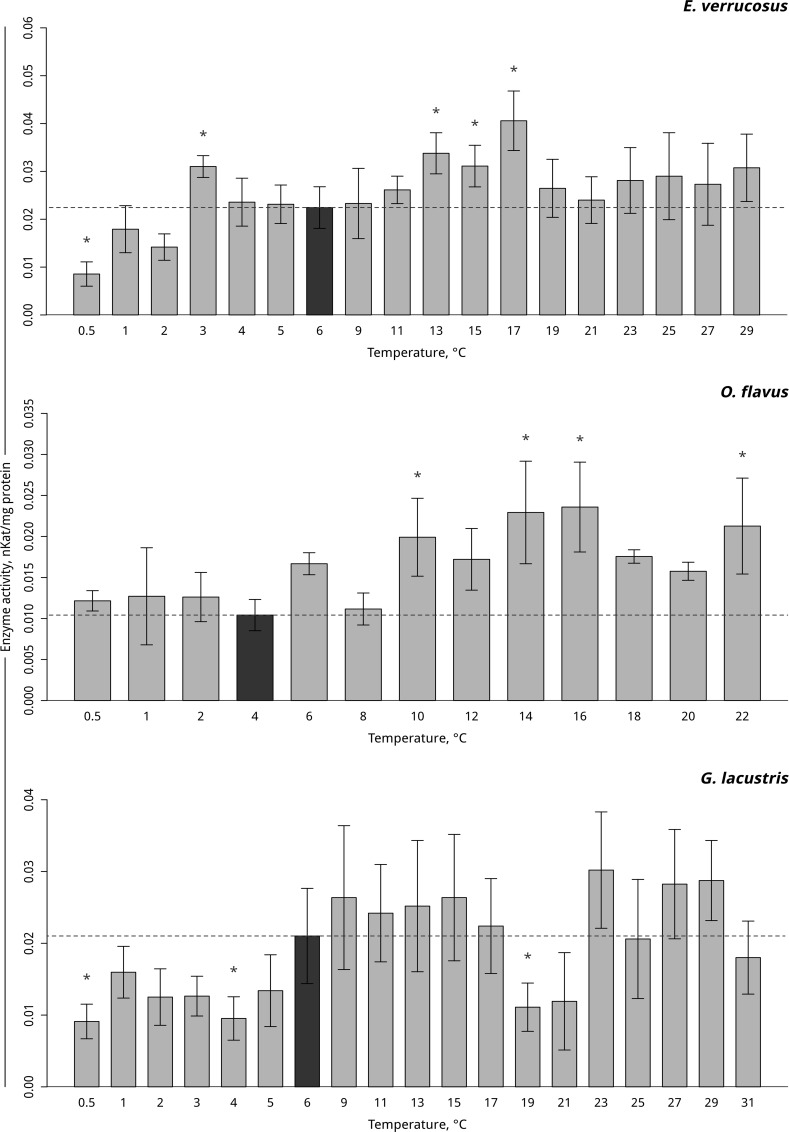
Peroxidase activity (in nKat/mg protein) in amphipod species during exposure to gradually changing temperatures. **The figure includes data on gradually increasing hypothermia and hyperthermia experiments.** *Indicates significant difference (p<0.05) from the control (*O*. *flavus*: 4°C; for *E*. *verrucosus*: 6°C and *G*. *lacustris*: 6°C). The dark grey columns and dotted line indicate the control level. Number of replicates: *O*. *flavus* (n_control_ = 6, n_exp._ = 4–5); *E*. *verrucosus* (n_control_ = 12, n_exp._ = 5–8) and *G*. *lacustris* (n_control_ = 13, n_exp._ = 5–8). Additional information presented in S4 Table.

**Fig 5:**
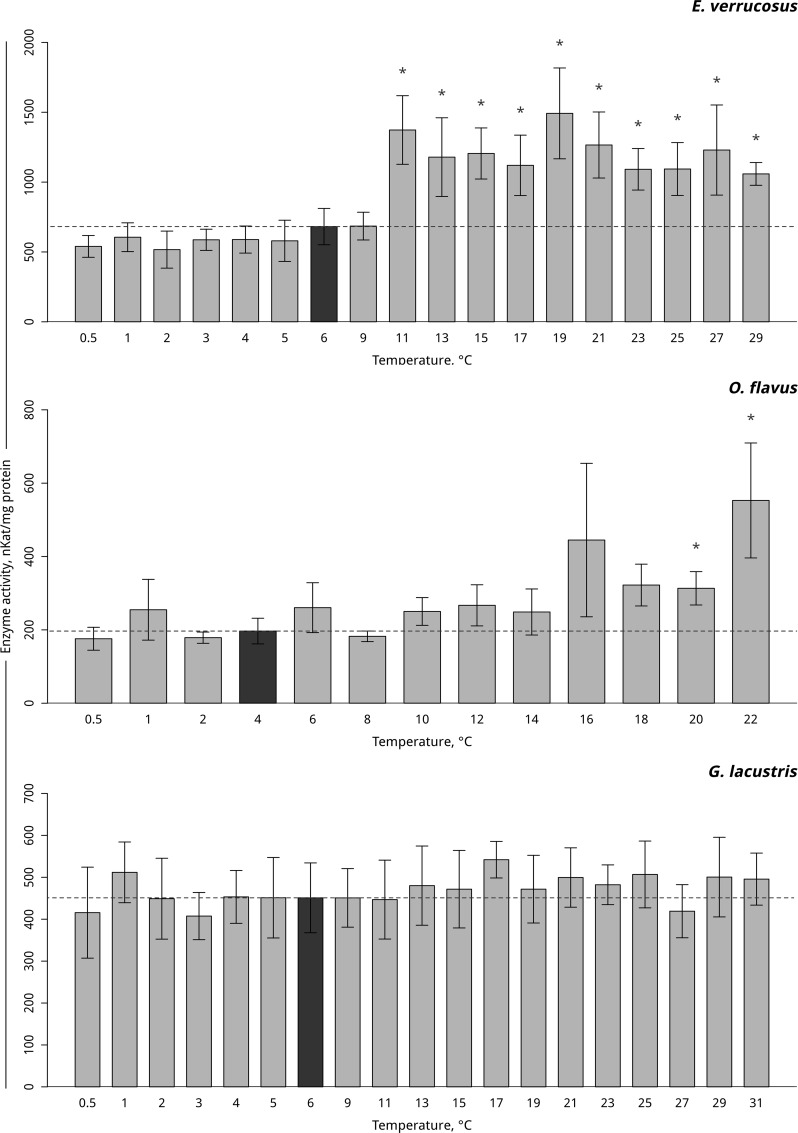
Catalase activity (in nKat/mg protein) in amphipods species during exposure to gradual temperature changes. **The figure compilation includes gradual hypothermia and hyperthermia experiments.** *Indicates significant difference (p<0.05) from the control (*O*. *flavus*: 4°C; for *E*. *verrucosus*: 6°C and *G*. *lacustris*: 6°C). The dark grey columns and dotted line indicate the control level. Number of replicates: *O*. *flavus* (n_control_ = 5, n_exp._ = 4–5); *E*. *verrucosus* (n_control_ = 12, n_exp._ = 5–7) and *G*. *lacustris* (n_control_ = 16, n_exp._ = 5–9). Additional information presented in S5 Table.

**Fig 6:**
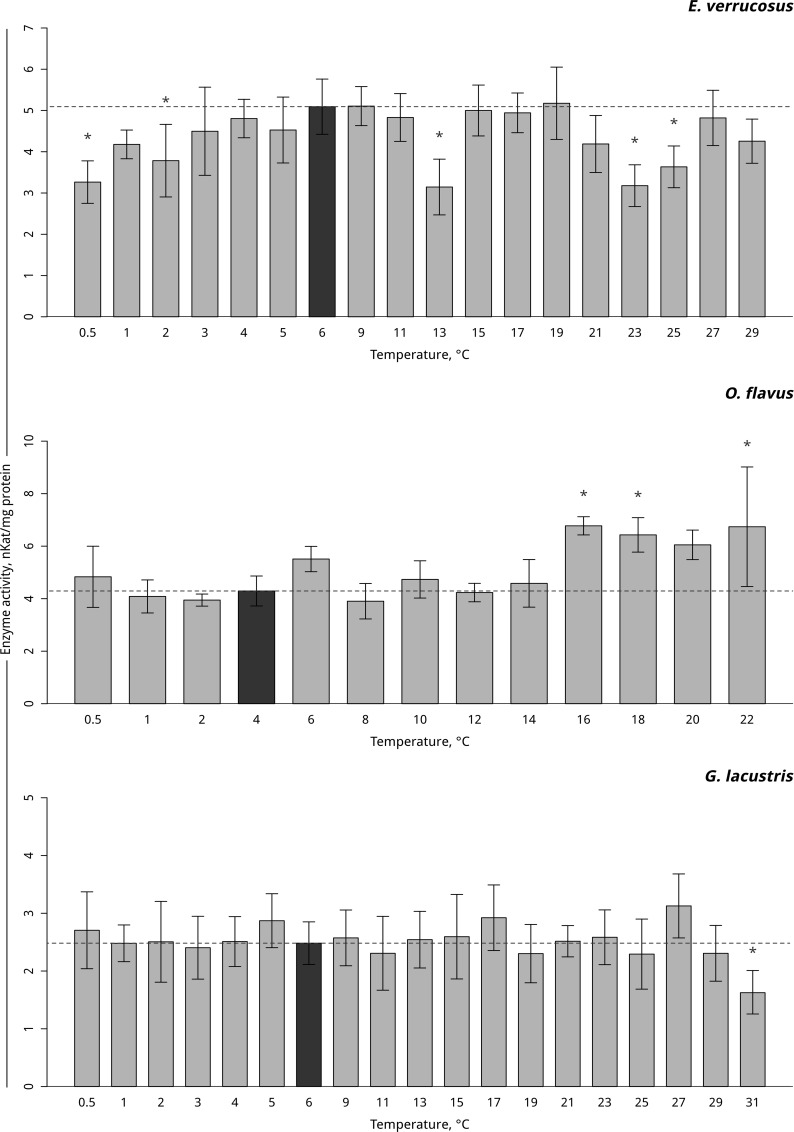
Glutathione S-transferase activity (in nKat/ mg protein) in amphipod species during exposure to gradual temperature changes. **The figure includes data obtained during increasing hypothermia and hyperthermia.** *Indicates a significant difference (p<0.05) from controls (*O*. *flavus*: 4°C; for *E*. *verrucosus*: 6°C and *G*. *lacustris*: 15°C). The dark grey columns and dotted line indicate the control level. *Indicates significant difference (p<0.05) from the control (*O*. *flavus*: 4°C; for *E*. *verrucosus*: 6°C and *G*. *lacustris*: 6°C). The dark grey columns and dotted line indicate the control level. Number of replicates: *O*. *flavus* (n_control_ = 6, n_exp._ = 4–5); *E*. *verrucosus* (n_control_ = 12, n_exp._ = 5–8) and *G*. *lacustris* (n_control_ = 16, n_exp._ = 5–9). Additional information presented in S6 Table.

Temperature-dependent antioxidative enzyme activities of the studied amphipods are presented in Figs 4–6 (S3–S6 Tables). A significant increase in peroxidase activity in the thermosensitive littoral Baikal species *E*. *verrucosus* occurred at 13°C– 17°C (p < 0.001) following by the return of activity to control values. As the temperature was gradually decreased, the activity of peroxidase increased at 3°C and subsequently decreased, which led to a significant decrease to below the control level at 0.5°C (Fig 4, S4 Table). Gradual temperature increase resulted in a significant increase in peroxidase activity in the thermosensitive deepwater Baikal species *O*. *flavus* starting from 10°C (p = 0.011) and further fluctuating slightly above the control level, with significantly higher activities at 14–16 and 22°C. The gradual temperature decrease did not elicit changes in peroxidase activity in this species (Fig 4, S4 Table). In the thermotolerant Holarctic species *G*. *lacustris*, gradual temperature increase resulted in a significant decline in peroxidase activity at 19°C (p = 0.001) followed by a return to the control level. Exposure to gradual temperature decrease led to a significant decline in peroxidase activity at 4°C (p = 0.01). A further decrease of the ambient temperature kept the level of peroxidase activity slightly below the control level, with a significant decrease at 0.5°C (p = 0.007) (Fig 4, S4 Table).

Gradual temperature increase resulted in a significant elevation in catalase activity in the thermosensitive littoral Baikal species *E*. *verrucosus*, starting from 11°C (p < 0.001). A further increase in temperature resulted a constant level of catalase activity, significantly higher than the control level (p < 0.001) (Fig 5, S5 Table). In the thermosensitive deepwater Baikal species *O*. *flavus*, gradual temperature increase resulted in a significant elevation in catalase activity, starting from 20°C (p = 0.03), which was followed by an increase at 22°C (p < 0.001) (Fig 5, S5 Table). No change in catalase activity was detected in the thermotolerant Holarctic species *G*. *lacustris* during the entire exposure. Gradual temperature decrease had no effect on catalase activity in all the three species (Fig 5, S5 Table).

A significant decrease in glutathione S-transferase activity in the thermosensitive littoral Baikal species *E*. *verrucosus* exposed to a gradual temperature elevation occurred at 13°C (p < 0.001), followed by an increase to the control level and subsequent significant decreases at 23 and 25°C (p < 0.001). Gradual temperature decrease resulted in significant decline in glutathione S-transferase activity at 2 and 0.5°C (p < 0.001) (Fig 6, S6 Table). Activity of glutathione S-transferase in the thermosensitive deepwater Baikal species *O*. *flavus* increased at 16°C– 22°C (p = 0.04). Gradual temperature decrease did not affect the activity of glutathione S-transferase in this species (Fig 6, S6 Table). No change in glutathione S-transferase activity was observed in the Holarctic species *G*. *lacustris* at any experimental temperature except 31°C, which resulted in decreased glutathione S-transferase activity (1.62 ± 0.32 nKat/mg protein (p = 0.06) (Fig 6, S6 Table).

#### Lactate content and lactate dehydrogenase activity

**Fig 7:**
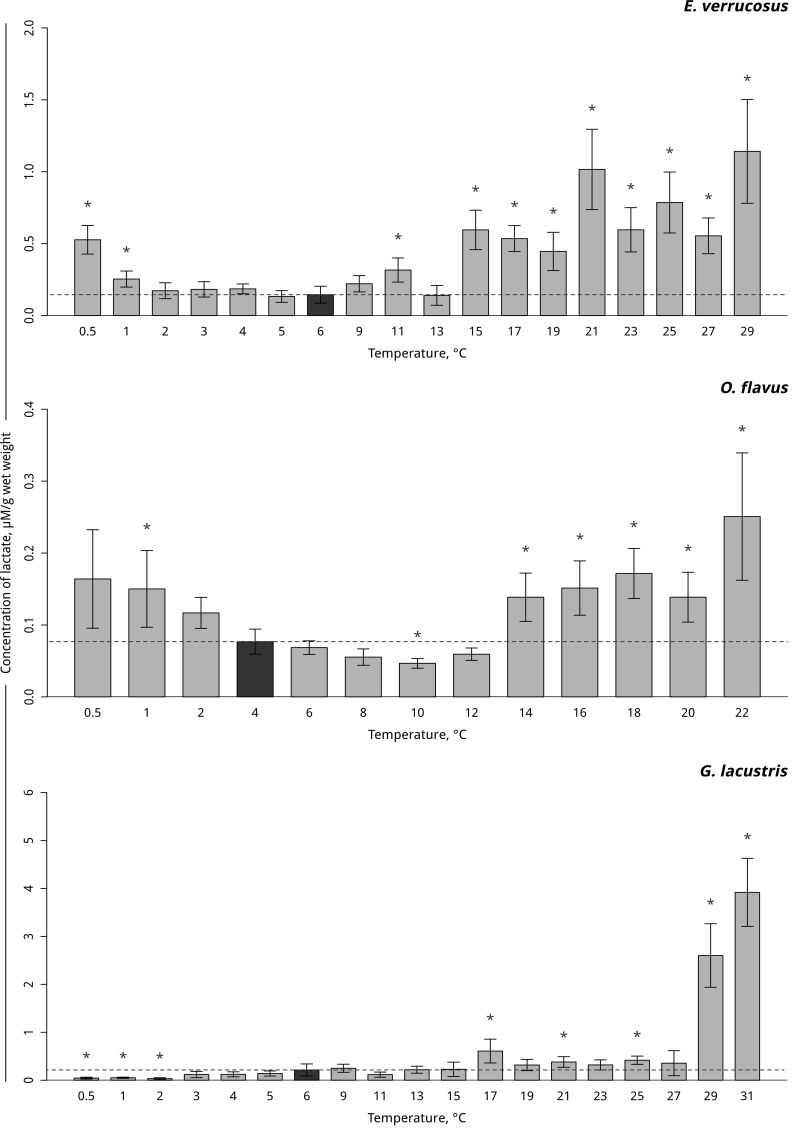
Lactate content (in μMol/g wet mass) in amphipods species during exposure to gradual temperature changes. **The figure includes data from hypothermia and hyperthermia experiments.** *Indicates significant difference (p<0.05) from the control (*O*. *flavus*: 4°C; for *E*. *verrucosus*: 6°C and *G*. *lacustris*: 6°C). The dark grey columns and dotted line indicate the control level. Number of replicates: *O*. *flavus* (n_control_ = 11, n_exp._ = 5–7); *E*. *verrucosus* (n_control_ = 13, n_exp._ = 5–6) and *G*. *lacustris* (n_control_ = 16, n_exp._ = 5–9). Additional information presented in S7 Table.

In the thermosensitive littoral Baikal species *E*. *verrucosus*, the lactate levels increased at 11°C (p = 0.001), with a short decline to the control level at 13°C and a subsequent significant increase beginning at 15°C and lasting until the end of exposure. Gradual temperature decrease resulted in a significant elevation in the lactate level beginning at 1°C and lasting until the end of exposure (p < 0.0001) (Fig 7, S7 Table). Gradual temperature increase led to a significant decrease in the lactate level at 10°C (p < 0.001) in the thermosensitive deepwater Baikal species *O*. *flavus*. Further increases in temperature resulted in the short restoration of the initial level of lactate with the onset of lactate accumulation at 14°C (p < 0.001). A significant accumulation of lactate during the gradual temperature decrease occurred at 1°C in this species (p = 0.022) (Fig 7, S7 Table). Significantly increased lactate was detected in the thermotolerant Holarctic species *G*. *lacustris* at 17°C (p < 0.001). Further increases in temperature resulted in slightly elevated lactate levels, with a manifold lactate accumulation after 29°C (p < 0.001). Decreasing the temperature to 2°C resulted in a significant decline in the lactate level in this species that lasted until the end of exposure (p = 0.005) (Fig 7, S7 Table).

**Fig 8:**
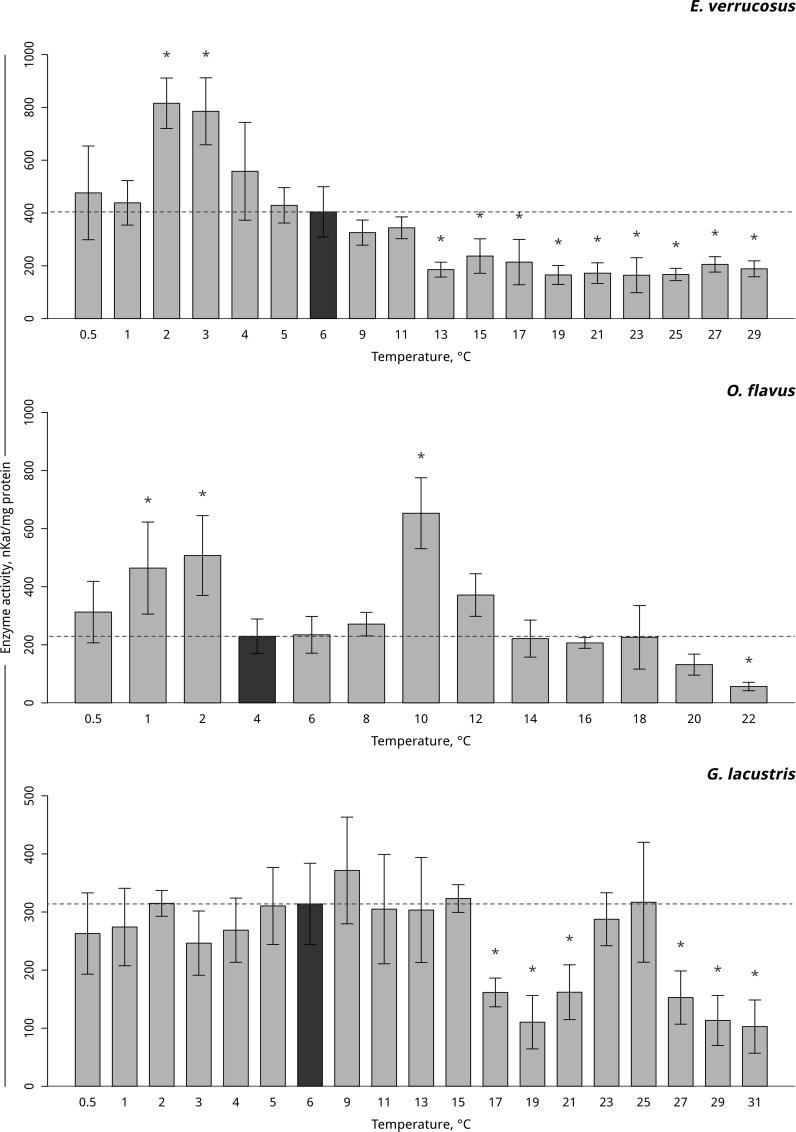
Lactate dehydrogenase activity (in nKat/ mg protein) in amphipods species during exposure to gradual temperature changes. **The figure includes data from hypothermia and hyperthermia experiments.** *Indicates significant difference (p<0.05) from the control (*O*. *flavus*: 4°C; for *E*. *verrucosus*: 6°C and *G*. *lacustris*: 6°C). The dark grey columns and dotted line indicate the control level. Number of replicates: *O*. *flavus* (n_control_ = 6, n_exp_. = 4–5); *E*. *verrucosus* (n_control_ = 13, n_exp._ = 6–9) and *G*. *lacustris* (n_control_ = 10, n_exp._ = 7–9). Additional information presented in S8 Table.

A gradual temperature increase resulted in a decrease of lactate dehydrogenase activity in the thermosensitive littoral Baikal species *E*. *verrucosus*, starting from 13°C (p < 0.001), and the activity further declined until the end of exposure. A gradual temperature decrease resulted in elevations in lactate dehydrogenase activity at 3 and 2°C (p < 0.001) with a consequent decline in activity to the end of exposure (Fig 8, S8 Table). In the thermosensitive deepwater Baikal species *O*. *flavus*, gradual temperature increase led to the significant elevation of lactate dehydrogenase activity at 10°C (p < 0.0001) with a following decline to the control level and a significant drop below the control level at 22°C (p < 0.001). Gradual temperature decrease resulted in elevations in lactate dehydrogenase activity at 2 and 1°C (p = 0.001) (Fig 8, S8 Table). Exposure of the thermotolerant Holarctic species *G*. *lacustris* to gradual temperature increase led to a decline in lactate dehydrogenase activity above 17°C (p < 0.001). The level of activity then briefly increased at 23 and 25°C to the control level and then again declined at 27°C and was significantly lower than the control level until the end of exposure (p < 0.001). Gradual temperature decrease did not influence the level of lactate dehydrogenase activity in *G*. *lacustris* (Fig 8, S8 Table).

## Discussion and Conclusion

Previous studies showed that thermal stress results in the activation of certain cellular and biochemical stress response mechanisms, such as Hsp70 and antioxidative systems, due to the degradation of cellular proteins and the accumulation of reactive oxygen species in various aquatic organisms [38–48], including Baikal amphipods and *G*. *lacustris* [18, 28, 49]. In addition, temperature increases led to activation of anaerobic metabolism in Baikal endemic amphipods [50, 51].

In the present study, gradual temperature increase caused elevated mortality rates in the investigated species. The determined LT50 and LT100 values confirmed the ranking of thermal resistance abilities of the studied amphipods, as follows: *O*. *flavus* (LT50: 23°C; LT100: 24°C) *< E*. *verrucosus* (LT50: 29.5°C; LT100: 31°C) *< G*. *lacustris* (LT50: 30.5°C; LT100: 33°C).

The effects of hyperthermia on cellular markers have previously been shown for the studied species [18, 28, 49] and can be explained by the functional role of the chosen markers. The increase in Hsp70 is a universal stress response to a variety of proteotoxic stresses, such as hyperthermia, which is connected to the molecular chaperone function of Hsp70. The main role of Hsp70 is to restore the native conformation and functional activity of damaged proteins [42, 52]. Thus, an increase in the Hsp70 level, elicited e.g., by heat and/or associated hypoxemia (cf. Pörtner [53]), indicates a stress defense to prevent protein degradation and to maintain protein homeostasis in the studied amphipod species. The molecular mechanisms of the Hsp70-related heat shock response in Baikal littoral amphipods have been described in our previous work [28]. The result of the present study confirmed the previously shown thermal-induced elevation in Hsp70 levels in Baikal amphipod *E*. *verrucosus* under gradual temperature increase. However, it was surprising to see the comparatively late onset of the Hsp70 induction in the thermosensitive deep-water *O*. *flavus*, which inhabits the zone with annually stable temperatures in the range of 3–4°C. The relatively high lethal temperature (LT50 = 25°C) and the late onset of the Hsp70 induction indicate a high thermal resistance of this species, possibly connected to its cross-resistance to a wide range of hydrostatic pressures, which requires a high activity of unspecific cellular defense systems (such as Hsp70). The induction of Hsp70 under high hydrostatic pressure has been shown previously [53–55]. Earlier sub-lethal constraints, as reported by L. Jakob et al. [56], still require investigation.

The increase in peroxidase and catalase activities possibly prevented the accumulation of free radicals produced under hyperthermic conditions [57], as has been shown for Baikal endemic amphipods [49, 58]. Peroxidases are responsible for the utilization of various hydroperoxides [57]. Peroxidase showed comparatively low activities (compared to the other two enzymes, catalase and glutathione S-transferase) in the studied amphipods, and no significant species-specific differences in enzyme activity were observed. The onset of significant alterations in peroxidase activity compared to control levels during the gradual temperature increase (first observed at 10, 13 and 19°C in *O*. *flavus*, *E*. *verrucosus* and *G*. *lacustris*, respectively) were correlated with the species-specific degree of thermotolerance. The decline in activity of this enzyme during hypothermia can indicate the decreased metabolic rate of the littoral Baikal species and *G*. *lacustris* at low temperatures, which began at 5°C in *G*. *lacustris* and at 0.5°C in *E*. *verrucosus*. The effects shown here are in accordance with the ecological parameters of the studied species. *E*. *verrucosus*, similar to several Baikal littoral amphipods, reproduces in winter when ambient temperatures of approximately 3°C prevail. In contrast, *G*. *lacustris* is a summer-reproducing species, which hibernates during the winter [23]. Unchanged peroxidase activity at low temperatures in the deep-water species *O*. *flavus* may indicate the absence of the ability of the antioxidative system to acclimate in order to cope with low temperatures because these species are never exposed to temperatures below 3.5°C in their environments.

Catalase is the essential enzyme required to utilize hydrogen peroxide in tissues and cells. Catalase was the most active of the enzymes studied here and showed the highest activities in both Baikal amphipod species, with the highest activity in the deep-water species *O*. *flavus*. Considering, that the Baikal species inhabits the highly oxygenated water of Lake Baikal (where the concentration of dissolved oxygen is up to 22 mg/L in the winter) [59], they may face higher oxidative pressure in this environment. The onset of the increase in catalase activities occurred in the Baikal species at 20°C in *O*. *flavus* and at 11°C in *E*. *verrucosus* but remained unchanged in *G*. *lacustris* during the entire exposure. Rising catalase activities indicate the occurrence of higher hydrogen peroxide levels in tissues and cells of the two Baikal species under exposure to hyperthermia. The lack of increased catalase activity in *G*. *lacustris* may be related to the higher thermotolerance of this species, which probably has other defense mechanisms (i.e., low molecular weight antioxidants) against hyperthermia, and the higher enzyme activity at elevated temperatures.

Glutathione S-transferase plays a key role in the removal of toxic metabolites, such as lipid hydroperoxides. This enzyme differed between the studied species: the activity of glutathione S-transferase was 1.5- to 2-fold higher in both Baikal species than in *G*. *lacustris*. The increased activity of this enzyme, as observed in *O*. *flavus* at 16°C, can therefore be considered a response to higher levels of products of secondary lipid peroxidation. The decreased activity of glutathione S-transferase in *E*. *verrucosus* at 0.5, 2, 13°C and 23–25°C and in *G*. *lacustris* at 31°C can be observed in the context of thermal acclimation; in addition to its role within the antioxidant system, glutathione S-transferase is also involved in the thermal inactivation of enzymes [57].

Lactate accumulation corresponded to decreased lactate dehydrogenase activities, which indicates an energy deficiency in the studied amphipod species upon exposure to pessimal temperatures [60, 61]. This pattern could be explained by energy demand in excess of aerobic energy production, causing a switch from aerobic to anaerobic metabolism. Lactate and lactate dehydrogenase are also major indicators of redox processes, which are reflected by the NAD/NADH balance. In this way, pyruvate converts into lactate to refill the NAD^+^ pool that is necessary for glycolysis flow. Thus, lactate is often shown as one of the main intermediates of energetic metabolites when species are exposed to non-optimal temperatures [62–64].

Numerous studies have shown that the decline in lactate dehydrogenase activity may be due to a lack of NADH or a depletion of glucose or glycogen content (see for example [65]); it may also occur during acclimation to elevated temperatures. In general, some observed changes in the levels of stress markers could be explained by a depression in the functional and physiological status of an organism; potential effects and reasons are summarized by Hochachka and Somero [4].

The maximum concentration of lactate and the minimum activity of lactate dehydrogenase were observed at the highest temperature, which indicates the direct effect of increasing temperature on oxygen metabolism. Accordingly, a clear negative correlation was observed between lactate and lactate dehydrogenase activity in the three species exposed to hyperthermic conditions (Table 2, S9 Table).

**Table 2:** Correlation analysis of lactate content and lactate dehydrogenase activity in *E*. *verrucosus*, *O*. *flavus* and *G*. *lacustris* exposed to temperature increase/decrease.

	*O*. *flavus*	*E*. *verrucosus*	*G*. *lacustris*
Hyperthermia (r)	-0.77	-0.85	-0.74
Hypothermia (r)	0.29	-0.08	-0.58

The species-specific peculiarities of the dynamics between changes in lactate content and lactate dehydrogenase activity may relate to differences in the stress-adaptation abilities between the Baikal and Holarctic *G*. *lacustris* species studied here and among species that inhabit different vertical zones (i.e., littoral and deep-water).

The increase in anaerobiosis with changing temperature can be explained by the concept of oxygen and capacity limitations of thermal tolerance [66], which states that each species (and even each life stage) has a limited thermal range of aerobic performance. The fluctuation in abiotic parameters from optimal ranges to pessimal zones leads to increases in anaerobic metabolism, oxidation processes and, consequently, oxidative stress, which is caused by the accumulation of reactive oxygen species [67].

As shown in the present study, the temperature ranges within which no changes were detected were not always the same for the parameters tested here. According to Liebig’s law, the physiological parameter performing optimally within the narrowest temperature range is the limiting factor for inducing a stress response [68, 69]. Among the tested stress markers tested here, the limiting factor for inducing a stress response was lactate, which reflected a zone of stable energetic metabolism within the temperature range of 2–8°C for *O*. *flavus*, 2–9°C for *E*. *verrucosus* and 3–15°C for *G*. *lacustris*. In line with oxygen and capacity limitation of thermal tolerance (OCLTT), lactate patterns indicate the limiting parameter in the three species, i.e., constraints in oxygen supply versus demand.

The upper temperatures of these ranges matched the thermal preference zones of the studied species. The thermal preference zone of *O*. *flavus* (4–8°C) determined in behavioral experiments corresponds to the zone of metabolite stability (2–8°C). The same pattern was observed for *G*. *lacustris* (thermal preference zone: 11–17°C) and its zone of metabolic stress marker stability (3–15°C). In the case of *E*. *verrucosus*, the two thermal ranges coincided almost perfectly (cellular stress markers: 4–9°C; thermal preference: 2–9°C).

Finally, our results demonstrate that the widths of the zones of cellular marker stability correlated with the thermotolerance of the studied amphipods species. The Holarctic species, *G*. *lacustris*, is characterized by a wider range of cellular marker stability (3°C -15°C) than that of the Baikal sublittoral species, *E*. *verrucosus* (2°C—9°C). In turn, the latter species is characterized by a wider range of cellular marker stability than the deep-water Baikal species, *O*. *flavus* (2°C—8°C).

Otherwise, the physiological basis of thermal behavior and thermal preference in crustaceans is not yet clear. As ectotherms, amphipods are unable to regulate their body temperature and need to avoid extreme temperatures. Specific thermal receptors are not yet found in crustaceans; however, it has been proposed that the peripheral nervous system can play a sensory role to provide the regulation of thermal behavior in crustaceans [70]. This regulation is especially important for the inhabitants of the littoral zone, where extreme temperature fluctuations often occur. In our study, we demonstrated that the deep-water Baikal species, *O*. *flavus*, has a relatively narrow zone of cellular marker stability but, as it was previously shown, it has a broad distribution within the thermal gradient in the thermopreference experiment, i.e., without a distinct peak compared with littoral species. It is possible that this species lacks thermal sensing and behavioral regulation due to long-term evolution under the stable isolated conditions of the Baikal bathyal zone. According to Pörtner [71], the thermal optimum for crustaceans is likely shaped by biochemical mechanisms eventually constraining the oxygen supply system, i.e., capacity limitations of ventilatory and circulatory organs. A previous 4-week study of ventilation and circulation, in relation to oxygen demand, showed that limitations in resting ventilation and oxygen consumption in *E*. *verrucosus* and *G*. *lacustris* are in accordance with the ranking of thermal tolerance determined in this study and previous studies of thermal preferences (for comparison see Jakob et al. [56]). In the present study, we demonstrate that the lactate and lactate dehydrogenase stability zones, which also correlate with thermal preference, can be determined even during short-term exposure.

We conclude that the thermal behavioral responses of the studied amphipods, which also reflect their distribution in the different vertical zones of Lake Baikal, are directly related to their metabolic processes at the cellular level. This relationship between behavioral reactions and stress response metabolism may have significant ecological consequences that result in a thermal zone-specific distribution (i.e., depths) of species and, as a result, decreased interspecific competition, which, in turn, increase the fitness of amphipod species in their respective environments.

## Supporting Information

S1 Table Raw data of distribution of *E*. *verrucosus* (n = 5), *O*. *flavus* (n = 4) and *G*. *lacustris* (n = 5) individuals in an experimental thermal gradient. (PDF)

S2 Table Raw data of mortality of *E*. *verrucosus* (n = 7), *O*. *flavus* (n = 11) and *G*. *lacustris* (n = 7) during exposure to a gradual temperature increase/decrease. (PDF)

S3 Table Raw data of Hsp70 levels in amphipod species during exposure to gradually changing temperatures. (PDF)

S4 Table Raw data of peroxidase activity (in nKat/ mg protein) in amphipod species during exposure to gradually changing temperatures. (PDF)

S5 Table Raw data of catalase activity (in nKat/ mg protein) in amphipods species during exposure to gradual temperature changes. (PDF)

S6 Table Raw data of glutathione S-transferase activity (in nKat/ mg protein) in amphipod species during exposure to gradual temperature changes. (PDF)

S7 Table Raw data of lactate content (in μMol/g wet mass) in amphipods species during exposure to gradual temperature changes. (PDF)

S8 Table Raw data of lactate dehydrogenase activity (in nKat/ mg protein) in amphipods species during exposure to gradual temperature changes. (PDF)

S9 Table Spearman’s rank correlation analysis. (PDF)
