# Supplementary material for: Thermal Preference Ranges Correlate with Stable Signals of Universal Stress Markers in Lake Baikal Endemic and Holarctic Amphipods
Source: PLoS One. 2016 Oct 5;11(10):e0164226. doi: 10.1371/journal.pone.0164226 (PMC5051968; doi:10.1371/journal.pone.0164226)
Supplement: S1 Table — (PDF) [file pone.0164226.s001.pdf]

S1 Table **Raw data of вistribution of *E. verrucosus* (n=5), *O. flavus* (n=4) and *G. lacustris* (n=5) individuals in an experimental thermal gradient** (Timofeyev et al. 2001, Timofeyev, Shatilina, 2007)

Species: *E. verrucosus*
Total number of Animals 500

	Temperature, °C									
	**3-4 °C**	**5-6°C**	**7-8°C**	**9-10°C**	**11-12°C**	**13-14°C**	**15-16°C**	**17-18°C**	**19-20°C**	**21-22°C**
**Raw data, %**	11.9	21.0	14.0	7.5	8.0	2.0	2.0	0.0	1.0	0.0
	19.3	33.2	23.2	14.5	9.9	6.6	6.0	4.0	1.9	0.0
	24.0	37.5	24.0	17.8	15.0	9.0	4.0	2.9	0.0	0.0
	13.0	25.8	20.0	12.0	12.0	5.2	3.0	4.1	5.0	1.0
	18.6	33.1	25.0	15.5	2.0	7.6	5.8	1.0	1.9	3.3
**N**	5.0	5.0	5.0	5.0	5.0	5.0	5.0	5.0	5.0	5.0
**MEAN**	17.4	30.1	21.2	13.5	9.4	6.1	4.2	2.4	2.0	0.9
**SD**	5.0	6.6	4.5	3.9	4.9	2.7	1.7	1.8	1.9	1.4

Species: *O. flavus*
Total number of Animals 312

	Temperature, °C								
	**3-4 °C**	**5-6°C**	**7-8°C**	**9-10°C**	**11-12°C**	**13-14°C**	**15-16°C**	**17-18°C**	**19-20°C**
**Raw data, %**	15.0	14.0	12.0	9.0	10.0	8.0	8.0	2.0	0.0
	19.0	18.7	11.0	12.2	12.0	10.0	8.0	3.0	2.0
	22.0	19.0	18.0	14.0	12.4	12.0	10.0	7.0	3.0
	24.0	25.0	20.0	18.0	16.2	14.0	12.0	7.0	3.0
**N**	4	4	4	4	4	4	4	4	4
**MEAN**	16.8	16.1	13.0	11.4	10.9	9.6	8.4	4.6	2.4
**SD**	7.9	7.8	6.3	5.3	4.5	3.8	3.0	2.3	1.5

Species: *G. lacustris*
Total number of Animals 475

	Temperature, °C								
	**3-4 °C**	**5-6°C**	**7-8°C**	**9-10°C**	**11-12°C**	**13-14°C**	**15-16°C**	**17-18°C**	**19-20°C**
**Raw data, %**	0.0	1.0	3.1	3.8	8.0	14.0	25.0	8.0	3.0
	1.0	1.7	3.2	4.2	10.1	16.0	28.7	11.8	4.2
	1.0	1.8	4.2	4.0	10.1	16.1	28.7	12.5	5.0
	1.7	2.0	6.9	5.5	12.1	19.2	34.3	14.1	5.1
	1.8	3.0	7.1	7.0	20.0	28.0	48.0	25.0	10.0
**N**	5	5	5	5	5	5	5	5	5
**MEAN**	1.1	1.9	4.9	4.9	12.1	18.7	33.0	14.3	5.5
**SD**	0.7	0.7	2.0	1.3	4.7	5.5	9.0	6.4	2.7

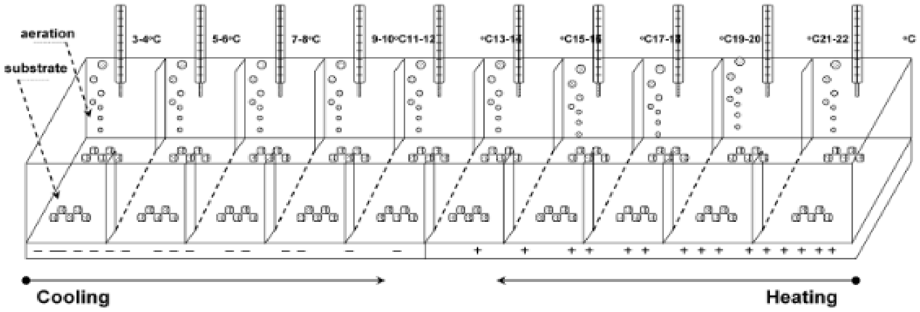

Fig. Gradient installation used for the experiments

References

Timofeyev MA, Shatilina JM, Stom DI. Attitude to temperature factor of some endemic amphipods from Lake Baikal and Holarctic G ammarus lacustris Sars , 1863 : A comparative experimental study. Arthropoda Sel. 2001;10: 93–101.

Timofeyev MA, Shatalina Z. Different preference reactions of three Lake Baikal endemic amphipods to temperature and oxygen are correlated with symbiotic life. Crustaceana. 2007;80: 129–138.
