# Supplementary material for: Thermal Preference Ranges Correlate with Stable Signals of Universal Stress Markers in Lake Baikal Endemic and Holarctic Amphipods
Source: PLoS One. 2016 Oct 5;11(10):e0164226. doi: 10.1371/journal.pone.0164226 (PMC5051968; doi:10.1371/journal.pone.0164226)
Supplement: S2 Table — (PDF) [file pone.0164226.s002.pdf]

S2 Table **Set of raw data of Mortality rate of *E. verrucosus. O. flavus and G. lacustris***

Species: *E. verrucosus*

Total number of animals *140*

Raw data, %

Temperature, °C	repl.1, % of dead	repl.2, % of dead	repl.3, % of dead	repl.4, % of dead	repl.5, % of dead	repl.6, % of dead	repl.7, % of dead	-	MEAN	SD
0.5	0.0	0.0	0.0	0.0	0.0	0.0	0.0		0.0	0.0
1	0.0	0.0	0.0	0.0	0.0	0.0	0.0		0.0	0.0
2	0.0	0.0	0.0	0.0	0.0	0.0	0.0		0.0	0.0
3	0.0	0.0	0.0	0.0	0.0	0.0	0.0		0.0	0.0
4	0.0	0.0	0.0	0.0	0.0	0.0	0.0		0.0	0.0
5	0.0	0.0	0.0	0.0	0.0	0.0	0.0		0.0	0.0
6	0.0	0.0	0.0	0.0	0.0	0.0	0.0		0.0	0.0
7	0.0	0.0	0.0	0.0	0.0	0.0	0.0		0.0	0.0
8	0.0	0.0	0.0	0.0	0.0	0.0	0.0		0.0	0.0
9	0.0	0.0	0.0	0.0	0.0	0.0	0.0		0.0	0.0
10	0.0	0.0	0.0	0.0	0.0	0.0	0.0		0.0	0.0
11	0.0	0.0	0.0	0.0	0.0	0.0	0.0		0.0	0.0
12	0.0	0.0	0.0	0.0	0.0	0.0	0.0		0.0	0.0
13	0.0	0.0	0.0	0.0	0.0	0.0	0.0		0.0	0.0
14	0.0	0.0	0.0	0.0	0.0	0.0	0.0		0.0	0.0
15	0.0	0.0	0.0	0.0	0.0	0.0	0.0		0.0	0.0
16	0.0	0.0	0.0	0.0	0.0	0.0	0.0		0.0	0.0
17	0.0	0.0	0.0	0.0	0.0	0.0	0.0		0.0	0.0
18	0.0	0.0	0.0	0.0	0.0	0.0	0.0		0.0	0.0
19	0.0	0.0	0.0	0.0	0.0	0.0	0.0		0.0	0.0
20	0.0	0.0	0.0	0.0	0.0	0.0	0.0		0.0	0.0
21	0.0	0.0	0.0	0.0	0.0	0.0	0.0		0.0	0.0
22	0.0	0.0	0.0	0.0	0.0	0.0	0.0		0.0	0.0
23	0.0	0.0	0.0	0.0	10.0	0.0	0.0		1.4	3.5
24	0.0	0.0	0.0	0.0	10.0	0.0	10.0		2.9	4.5
25	0.0	0.0	0.0	0.0	10.0	0.0	10.0		2.9	4.5
26	0.0	0.0	0.0	0.0	10.0	0.0	10.0		2.9	4.5
27	0.0	0.0	0.0	0.0	10.0	0.0	10.0		2.9	4.5
28	10.0	10.0	10.0	0.0	10.0	0.0	20.0		8.6	6.4
29	20.0	10.0	30.0	10.0	10.0	20.0	40.0		20.0	10.7
30	50.0	50.0	50.0	50.0	50.0	30.0	80.0		51.4	13.6
31	100.0	100.0	100.0	100.0	100.0	100.0	100.0		100.0	0.0

Species: *O. flavus*

Total number of animals *110*

Raw data, %

Temperature, °C	repl.1, % of dead	repl.2, % of dead	repl.3, % of dead	repl.4, % of dead	repl.5, % of dead	repl.6, % of dead	repl.7, % of dead	repl.8, % of dead	repl.9, % of dead	repl.10, % of dead	repl.11, % of dead	-	MEAN	SD
0.5	0.0	0.0	0.0	0.0	0.0	0.0	0.0	0.0	0.0	0.0	0.0		0.0	0.0
1	0.0	0.0	0.0	0.0	0.0	0.0	0.0	0.0	0.0	0.0	0.0		0.0	0.0
2	0.0	0.0	0.0	0.0	0.0	0.0	0.0	0.0	0.0	0.0	0.0		0.0	0.0
3	0.0	0.0	0.0	0.0	0.0	0.0	0.0	0.0	0.0	0.0	0.0		0.0	0.0
4	0.0	0.0	0.0	0.0	0.0	0.0	0.0	0.0	0.0	0.0	0.0		0.0	0.0
5	0.0	0.0	0.0	0.0	0.0	0.0	0.0	0.0	0.0	0.0	0.0		0.0	0.0
6	0.0	0.0	0.0	0.0	0.0	0.0	0.0	0.0	0.0	0.0	0.0		0.0	0.0
7	0.0	0.0	0.0	0.0	0.0	0.0	0.0	0.0	0.0	0.0	0.0		0.0	0.0
8	0.0	0.0	0.0	0.0	0.0	0.0	0.0	0.0	0.0	0.0	0.0		0.0	0.0
9	0.0	0.0	0.0	0.0	0.0	0.0	0.0	0.0	0.0	0.0	0.0		0.0	0.0
10	0.0	0.0	0.0	0.0	0.0	0.0	0.0	0.0	0.0	0.0	0.0		0.0	0.0
11	0.0	0.0	0.0	0.0	0.0	0.0	0.0	0.0	0.0	0.0	0.0		0.0	0.0
12	0.0	0.0	0.0	0.0	0.0	0.0	0.0	0.0	0.0	0.0	0.0		0.0	0.0
13	0.0	0.0	0.0	0.0	0.0	0.0	0.0	0.0	0.0	0.0	0.0		0.0	0.0
14	0.0	0.0	0.0	0.0	0.0	0.0	0.0	0.0	0.0	0.0	0.0		0.0	0.0
15	0.0	0.0	0.0	0.0	0.0	0.0	0.0	0.0	0.0	0.0	0.0		0.0	0.0
16	0.0	0.0	0.0	0.0	0.0	0.0	0.0	0.0	0.0	0.0	0.0		0.0	0.0
17	0.0	0.0	0.0	0.0	10.0	0.0	0.0	0.0	0.0	0.0	0.0		0.9	2.9
18	0.0	0.0	0.0	0.0	10.0	0.0	0.0	0.0	0.0	0.0	0.0		0.9	2.9
19	0.0	0.0	0.0	0.0	10.0	0.0	0.0	0.0	0.0	0.0	0.0		0.9	2.9
20	0.0	0.0	0.0	0.0	10.0	0.0	0.0	0.0	0.0	0.0	0.0		0.9	2.9
21	0.0	0.0	0.0	0.0	10.0	0.0	0.0	10.0	0.0	0.0	0.0		1.8	3.9
22	0.0	11.1	0.0	0.0	10.0	0.0	0.0	20.0	0.0	0.0	0.0		3.7	6.5
23	0.0	11.1	0.0	0.0	10.0	10.0	0.0	20.0	0.0	0.0	0.0		4.6	6.6
24	80.0	11.1	0.0	0.0	20.0	10.0	0.0	20.0	0.0	0.0	0.0		12.8	22.6
25	100.0	22.2	0.0	0.0	40.0	70.0	45.5	20.0	11.1	18.2	20.0		31.5	29.2
26		100.0	100.0	88.9	80.0	80.0	90.9	90.0	66.7	54.5	80.0		83.1	13.5
26.5				100.0	100.0	100.0	100.0	100.0	77.8	100.0	90.0		96.0	7.6
27									100.0		100.0		100.0	0.0

Species: *G. lacustris*

Total number of animals *140*

Raw data, %

Temperature, °C	repl.1, % of dead	repl.2, % of dead	repl.3, % of dead	repl.4, % of dead	repl.5, % of dead	repl.6, % of dead	repl.7, % of dead	-	MEAN	SD
0.5	0.0	0.0	0.0	0.0	0.0	0.0	0.0		0.0	0.0
1	0.0	0.0	0.0	0.0	0.0	0.0	0.0		0.0	0.0
2	0.0	0.0	0.0	0.0	0.0	0.0	0.0		0.0	0.0
3	0.0	0.0	0.0	0.0	0.0	0.0	0.0		0.0	0.0
4	0.0	0.0	0.0	0.0	0.0	0.0	0.0		0.0	0.0
5	0.0	0.0	0.0	0.0	0.0	0.0	0.0		0.0	0.0
6	0.0	0.0	0.0	0.0	0.0	0.0	0.0		0.0	0.0
7	0.0	0.0	0.0	0.0	0.0	0.0	0.0		0.0	0.0
8	0.0	0.0	0.0	0.0	0.0	0.0	0.0		0.0	0.0
9	0.0	0.0	0.0	0.0	0.0	0.0	0.0		0.0	0.0
10	0.0	0.0	0.0	0.0	0.0	0.0	0.0		0.0	0.0
11	0.0	0.0	0.0	0.0	0.0	0.0	0.0		0.0	0.0
12	0.0	0.0	0.0	0.0	0.0	0.0	0.0		0.0	0.0
13	0.0	0.0	0.0	0.0	0.0	0.0	0.0		0.0	0.0
14	0.0	0.0	0.0	0.0	0.0	0.0	0.0		0.0	0.0
15	0.0	0.0	0.0	0.0	0.0	0.0	0.0		0.0	0.0
16	0.0	0.0	0.0	0.0	0.0	0.0	0.0		0.0	0.0
17	0.0	0.0	0.0	0.0	0.0	0.0	0.0		0.0	0.0
18	0.0	0.0	0.0	0.0	0.0	0.0	0.0		0.0	0.0
19	0.0	0.0	0.0	0.0	0.0	0.0	0.0		0.0	0.0
20	0.0	0.0	0.0	0.0	0.0	0.0	0.0		0.0	0.0
21	0.0	0.0	0.0	0.0	0.0	0.0	0.0		0.0	0.0
22	0.0	0.0	0.0	0.0	0.0	0.0	0.0		0.0	0.0
23	5.0	0.0	0.0	0.0	0.0	0.0	0.0		0.7	1.7
24	5.0	5.0	0.0	0.0	0.0	0.0	0.0		1.4	2.3
25	5.0	5.0	0.0	0.0	0.0	0.0	5.0		2.1	2.5
26	5.0	5.0	0.0	0.0	7.0	0.0	5.0		3.1	2.8
27	5.0	15.0	0.0	5.0	7.0	5.0	5.0		6.0	4.2
28	8.0	18.0	0.0	5.0	13.0	11.0	10.0		9.3	5.3
29	10.0	20.0	0.0	5.0	20.0	38.0	10.0		14.7	11.7
30	25.0	20.0	0.0	16.0	20.0	59.0	22.0		23.1	16.5
31	35.0	65.0	26.0	63.0	47.0	64.0	49.0		49.9	14.1
32	75.0	80.0	58.0	79.0	60.0	79.0	58.0		69.9	9.8
33	100.0	100.0	100.0	100.0	100.0	100.0	100.0		100.0	0.0
