# Supplementary material for: Thermal Preference Ranges Correlate with Stable Signals of Universal Stress Markers in Lake Baikal Endemic and Holarctic Amphipods
Source: PLoS One. 2016 Oct 5;11(10):e0164226. doi: 10.1371/journal.pone.0164226 (PMC5051968; doi:10.1371/journal.pone.0164226)
Supplement: S3 Table — (PDF) [file pone.0164226.s003.pdf]

S3 Table **Set of raw data of Hsp70 levels in amphipod species during exposure to gradually changing temperatures.**

MW - molecular weight, PC - positive control, Arb.un - arbitrary units
PC^{HSP70} - positive control for heat shock protein 70
PC^{ACT} - positive control for actin

Species: *E.verrucosus*
Total number of animals 184
Number of animals/analisys 2

Temperature, °C	0.5	1	2	3	4	5	6	9	11	13	15	17	19	21	23	25	27	29
Raw data, arb.un	23.3	17.5	29.9	39.8	88.6	86.6	71.0	73.0	61.4	75.0	36.0	33.9	18.4	9.1	46.3	14.1	12.5	10.0
	32.0	36.2	63.0	53.0	100.0	105.0	79.9	83.1	305.2	170.0	80.0	63.9	70.0	65.0	100.0	56.7	60.0	40.1
	44.0	50.0	64.4	60.1	106.9	110.0	85.0	90.0	345.2	200.0	150.4	76.0	82.7	73.2	116.2	60.4	68.5	49.8
	53.4	55.0	68.0	63.0	108.0	111.2	98.0	110.0	408.0	216.0	180.0	78.2	90.0	80.0	120.0	68.7	74.1	77.9
	78.8	92.5	97.3	99.2	116.5	114.8	102.0	152.2	469.7	282.0	307.9	119.1	94.2	100.4	126.8	127.2	102.0	
							115.0											
							120.9											
							128.3											
N	5.0	5.0	5.0	5.0	5.0	5.0	8.0	5.0	5.0	5.0	5.0	5.0	5.0	5.0	5.0	5.0	5.0	4.0
MEAN	46.3	50.2	64.5	63.0	104.0	105.5	100.0	101.7	317.9	188.6	150.9	74.2	71.0	65.5	101.9	65.4	63.4	44.5
SD	19.2	24.8	21.4	19.8	9.3	10.0	19.2	28.0	139.9	67.6	93.5	27.5	27.6	30.6	29.1	36.2	29.1	24.3

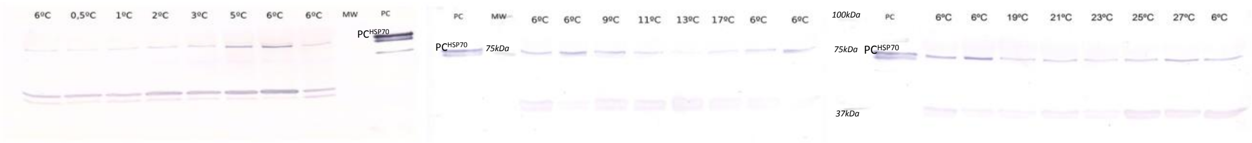

MW - molecular weight, PC - positive control, Arb.un - arbitrary units

Species: *O. flavus*
Total number of animals 104
Number of animals/analisys 2

Temperature, °C	0.5	1	2	4	6	8	10	12	14	16	18	20	22
Raw data, arb.un	101.6	111.8	75.0	72.3	94.7	115.9	132.0	118.1	105.2	140.2	141.5	210.0	121.8
	114.9	120.5	80.0	94.6	101.9	124.0	144.1	110.2	135.0	142.6	142.2	219.3	132.4
	140.1	133.6	136.2	109.0	105.0	128.1	148.3	184.8	140.5	151.4	257.9	224.0	149.8
	158.1	134.1	137.2	124.1	112.1	138.9	180.5	186.9	156.0	153.2	258.7	239.0	160.9
N	4.0	4.0	4.0	4.0	4.0	4.0	4.0	4.0	4.0	4.0	4.0	4.0	4.0
MEAN	128.7	125.0	107.1	100.0	103.4	126.7	151.2	150.0	134.1	146.9	200.1	223.1	141.2
SD	21.9	9.4	29.7	19.1	6.2	8.3	17.9	36.0	18.4	5.5	58.2	10.5	15.1

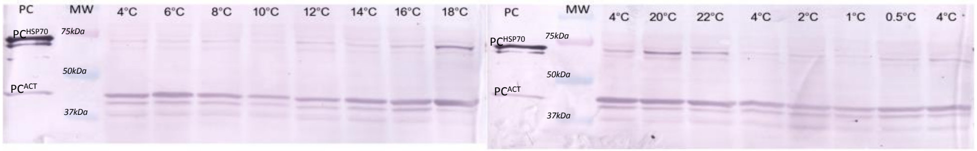

MW - molecular weight, PC - positive control, Arb.un - arbitrary units

Species: *G. lacustris*
Total number of animals 695
Number of animals/analisys 5

Temperature, °C	0.5	1	2	3	4	5	6	9	11	13	15	17	19	21	23	25	27	29	31
Raw data, arb.un	2.3	10.0	16.5	16.6	66.1	74.0	73.0	82.0	24.9	79.7	50.0	138.0	108.7	36.6	41.0	79.6	96.1	66.0	322.3
	24.2	14.7	20.0	102.0	180.0	146.0	97.6	85.6	123.1	200.0	61.9	139.8	111.9	130.0	85.0	98.0	150.0	100.1	575.0
	25.8	32.2	130.2	150.0	190.0	152.0	100.0	100.0	170.0	222.8	123.6	139.9	191.5	379.1	95.6	110.0	156.0	181.3	960.5
	26.3	44.9	170.4	220.0	217.8	165.4	108.2	105.0	190.0	432.2	210.0	378.5	231.0	750.0	200.0	120.0	170.0	270.0	1168.2
	49.3	127.5	206.1	261.4	278.9	195.7	121.2	127.4	378.7	481.6	376.1	492.7	314.3	975.0	354.4	172.1	228.0	321.9	1380.2
N	5.0	5.0	5.0	5.0	5.0	5.0	5.0	5.0	5.0	5.0	5.0	5.0	5.0	5.0	5.0	5.0	5.0	5.0	5.0
MEAN	25.6	45.9	108.6	150.0	186.6	146.6	100.0	100.0	177.3	283.3	164.3	257.8	191.5	454.1	155.2	116.0	160.0	187.9	881.3
SD	16.6	47.7	86.8	96.7	77.6	44.9	17.7	18.1	129.4	168.5	134.3	167.3	86.3	401.2	125.7	34.8	47.2	108.8	431.2

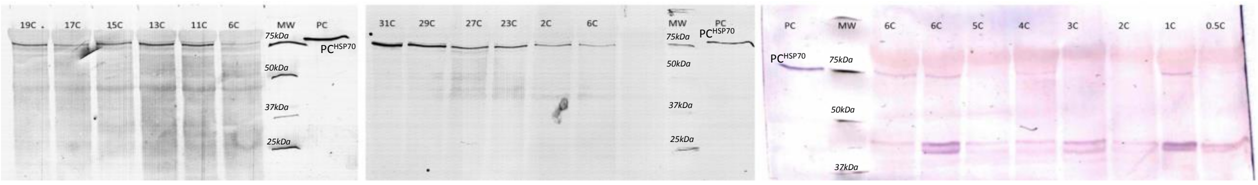

MW - molecular weight, PC - positive control, Arb.un - arbitrary units
