# Supplementary material for: Thermal Preference Ranges Correlate with Stable Signals of Universal Stress Markers in Lake Baikal Endemic and Holarctic Amphipods
Source: PLoS One. 2016 Oct 5;11(10):e0164226. doi: 10.1371/journal.pone.0164226 (PMC5051968; doi:10.1371/journal.pone.0164226)
Supplement: S4 Table — (PDF) [file pone.0164226.s004.pdf]

S4 Table **Set of raw data of peroxidase activity (in nKat/ mg protein) in amphipod species during exposure to gradually changing temperatures.**

Species: *E.verrucosus*

Total number of animals 110
Number of animals/analisys 1

**Temperature, °C**	**0.5**	**1**	**2**	**3**	**4**	**5**	**6**	**9**	**11**	**13**	**15**	**17**	**19**	**21**	**23**	**25**	**27**	**29**
**Raw data, nKat/mg prot**	0.004	0.012	0.010	0.028	0.018	0.018	0.016	0.010	0.023	0.028	0.025	0.031	0.015	0.017	0.017	0.015	0.016	0.020
	0.007	0.013	0.014	0.029	0.019	0.020	0.017	0.017	0.023	0.031	0.030	0.036	0.025	0.022	0.027	0.026	0.021	0.027
	0.009	0.020	0.015	0.031	0.020	0.022	0.019	0.022	0.027	0.031	0.031	0.040	0.028	0.023	0.028	0.031	0.033	0.034
	0.009	0.020	0.016	0.031	0.027	0.025	0.019	0.024	0.028	0.036	0.032	0.040	0.028	0.023	0.030	0.034	0.033	0.035
	0.009	0.024	0.017	0.033	0.027	0.027	0.020	0.024	0.029	0.037	0.037	0.041	0.030	0.029	0.036	0.039	0.034	0.038
	0.011			0.035	0.030	0.028	0.021	0.026		0.039		0.045	0.033	0.030				
	0.012						0.023	0.031				0.051						
							0.026	0.033										
							0.026											
							0.027											
							0.027											
							0.028											
**N**	7.0	5.0	5.0	6.0	6.0	6.0	12.0	8.0	5.0	6.0	5.0	7.0	6.0	6.0	5.0	5.0	5.0	5.0
**MEAN**	0.01	0.02	0.01	0.03	0.02	0.02	0.02	0.02	0.03	0.03	0.03	0.04	0.03	0.02	0.03	0.03	0.03	0.03
**SD**	0.00	0.00	0.00	0.00	0.01	0.00	0.00	0.01	0.00	0.00	0.00	0.01	0.01	0.00	0.01	0.01	0.01	0.01

Species: *O. flavus*
Total number of animals 192
Number of animals/analisys 3

**Temperature, °C**	**0.5**	**1**	**2**	**4**	**6**	**8**	**10**	**12**	**14**	**16**	**18**	**20**	**22**
**Raw data, nKat/mg prot**	0.013	0.018	0.013	0.014	0.017	0.012	0.026	0.024	0.032	0.030	0.018	0.017	0.025
	0.011	0.007	0.015	0.011	0.018	0.013	0.019	0.015	0.015	0.017	0.017	0.015	0.022
	0.012	0.015	0.009	0.011	0.015	0.009	0.017	0.015	0.024	0.023	0.018	0.016	0.013
	0.011	0.010	0.010	0.009	0.016	0.011	0.022	0.016	0.022	0.024	0.018	0.015	0.025
	0.012	0.009		0.009	0.018	0.009	0.014	0.015	0.021	0.024	0.017	0.016	
				0.009									
**N**	5.0	5.0	4.0	6.0	5.0	5.0	5.0	5.0	5.0	5.0	5.0	5.0	4.0
**MEAN**	0.012	0.012	0.012	0.010	0.017	0.011	0.020	0.017	0.023	0.024	0.018	0.016	0.021
**SD**	0.001	0.005	0.003	0.002	0.001	0.002	0.005	0.004	0.006	0.005	0.001	0.001	0.006

Species: *G. lacustris*
Total number of animals 660
Number of animals/analisys 5

**Temperature, °C**	**0.5**	**1**	**2**	**3**	**4**	**5**	**6**	**9**	**11**	**13**	**15**	**17**	**19**	**21**	**23**	**25**	**27**	**29**	**31**
**Raw data, nKat/mg prot**	0.007	0.011	0.008	0.008	0.006	0.008	0.014	0.011	0.018	0.015	0.019	0.012	0.008	0.003	0.017	0.014	0.021	0.023	0.014
	0.007	0.015	0.009	0.011	0.007	0.009	0.015	0.017	0.020	0.015	0.023	0.018	0.008	0.005	0.026	0.014	0.023	0.025	0.015
	0.007	0.016	0.013	0.012	0.009	0.009	0.016	0.022	0.021	0.021	0.023	0.020	0.008	0.010	0.028	0.016	0.023	0.026	0.016
	0.010	0.017	0.015	0.012	0.009	0.012	0.016	0.024	0.022	0.025	0.023	0.023	0.010	0.010	0.031	0.016	0.026	0.028	0.017
	0.011	0.021	0.017	0.012	0.012	0.017	0.017	0.029	0.022	0.030	0.026	0.026	0.014	0.017	0.033	0.019	0.027	0.032	0.017
	0.012			0.016	0.014	0.019	0.017	0.030	0.028	0.030	0.026	0.027	0.014	0.017	0.033	0.032	0.039	0.038	0.027
				0.016		0.019	0.019	0.038	0.038	0.040	0.046	0.032	0.016	0.021	0.043	0.033	0.040		
							0.020	0.040											
							0.021												
							0.026												
							0.028												
							0.030												
							0.035												
**N**	6.0	5.0	5.0	7.0	6.0	7.0	13.0	8.0	7.0	7.0	7.0	7.0	7.0	7.0	7.0	7.0	7.0	6.0	6.0
**MEAN**	0.009	0.016	0.013	0.013	0.010	0.013	0.021	0.026	0.024	0.025	0.026	0.022	0.011	0.012	0.030	0.021	0.028	0.029	0.018
**SD**	0.002	0.004	0.004	0.003	0.003	0.005	0.007	0.010	0.007	0.009	0.009	0.007	0.003	0.007	0.008	0.008	0.008	0.006	0.005
