# Supplementary material for: Thermal Preference Ranges Correlate with Stable Signals of Universal Stress Markers in Lake Baikal Endemic and Holarctic Amphipods
Source: PLoS One. 2016 Oct 5;11(10):e0164226. doi: 10.1371/journal.pone.0164226 (PMC5051968; doi:10.1371/journal.pone.0164226)
Supplement: S5 Table — (PDF) [file pone.0164226.s005.pdf]

S5 Table **Set of raw data of catalase activity (in nKat/ mg protein) in amphipods species during exposure to gradual temperature changes.**

Species: *E.verrucosus*
Total number of animals 110
Number of animals/analisys 1

**Temperature, °C**	**0.5**	**1.0**	**2.0**	**3.0**	**4.0**	**5.0**	**6.0**	**9.0**	**11.0**	**13.0**	**15.0**	**17.0**	**19.0**	**21.0**	**23.0**	**25.0**	**27.0**	**29.0**
**Raw data, nKat/mg prot**	445.5	466.1	399.0	483.5	482.9	429.6	481.4	557.6	1037.7	799.4	1074.8	851.1	1109.6	990.8	865.1	811.0	815.2	950.1
	457.3	527.5	400.2	540.2	487.3	482.5	493.2	640.1	1201.0	1055.5	1128.3	935.9	1311.9	1098.0	1077.0	1002.9	1145.3	999.7
	532.1	650.6	468.1	545.8	535.1	535.5	511.7	643.3	1348.1	1109.3	1142.4	1011.4	1360.1	1167.8	1120.6	1144.2	1225.8	1084.0
	547.7	688.1	641.3	631.4	673.2	535.5	645.6	666.6	1352.4	1112.1	1151.4	1066.8	1391.7	1241.3	1120.7	1243.9	1244.7	1113.9
	553.3	695.2	675.0	634.5	674.8	656.2	654.8	682.3	1596.8	1388.3	1527.7	1199.7	1829.3	1491.6	1273.9	1266.4	1716.4	1145.6
	561.3			686.4	678.7	838.8	671.1	728.1	1701.9	1608.3		1315.3	1948.8	1604.1				
	681.3						685.2	877.5				1459.4						
							787.7											
							805.1											
							807.1											
							814.6											
							821.0											
**N**	7.0	5.0	5.0	6.0	6.0	6.0	12.0	7.0	6.0	6.0	5.0	7.0	6.0	6.0	5.0	5.0	5.0	5.0
**MEAN**	539.8	605.5	516.7	587.0	588.7	579.7	681.5	685.1	1373.0	1178.8	1204.9	1119.9	1491.9	1265.6	1091.5	1093.7	1229.5	1058.6
**SD**	72.2	92.1	118.6	69.2	88.5	134.7	124.5	92.0	224.1	257.2	163.6	200.2	296.8	215.8	131.5	169.2	288.5	72.8

Species: *O. flavus*
Total number of animals 195
Number of animals/analisys 3

**Temperature, °C**	**0.5**	**1**	**2**	**4**	**6**	**8**	**10**	**12**	**14**	**16**	**18**	**20**	**22**
**Raw data, nKat/mg prot**	126.6	157.0	152.0	134.9	273.9	164.0	195.6	213.9	185.5	206.6	243.0	243.7	378.7
	175.7	249.9	178.4	179.8	157.9	180.0	242.9	235.9	225.9	440.0	320.0	313.2	462.8
	180.0	250.0	186.0	200.0	262.8	182.0	259.0	260.8	240.0	445.0	329.2	315.0	671.6
	185.4	270.9	186.9	213.0	280.0	192.1	269.0	275.0	251.0	460.0	340.0	336.1	698.3
	210.8	346.4	189.9	226.3	328.0	193.8	284.5	348.8	340.9	672.9	375.6	358.8	
				226.3									
**N**	5.0	5.0	5.0	6.0	5.0	5.0	5.0	5.0	5.0	5.0	5.0	5.0	4.0
**MEAN**	175.7	254.8	178.6	196.7	260.5	182.4	250.2	266.9	248.7	444.9	321.6	313.4	552.8
**SD**	27.4	60.4	13.8	32.0	55.9	10.7	30.5	46.0	51.2	147.7	43.6	38.6	135.7

Species: *G. lacustris*
Total number of animals 670
Number of animals/analisys 5

**Temperature, °C**	**0.5**	**1**	**2**	**3**	**4**	**5**	**6**	**9**	**11**	**13**	**15**	**17**	**19**	**21**	**23**	**25**	**27**	**29**	**31**
**Raw data, nKat/mg prot**	310.8	414.5	326.9	326.9	377.8	356.8	275.8	329.0	346.1	320.0	381.1	452.0	394.6	416.9	418.1	398.0	314.1	395.8	401.7
	329.6	503.5	397.5	366.2	391.3	391.1	380.4	428.1	348.0	395.6	391.2	536.0	399.6	446.3	440.1	417.6	357.6	416.0	463.3
	329.6	507.4	426.6	366.2	428.9	391.1	392.7	429.2	400.7	473.6	436.6	540.4	410.8	467.8	473.4	475.3	414.3	436.3	490.2
	341.6	514.9	545.4	394.8	493.7	392.5	398.1	432.1	406.0	483.7	443.0	546.9	434.6	502.6	475.8	527.1	437.6	559.8	495.1
	341.6	618.7	548.5	418.6	494.8	412.1	400.2	443.9	504.2	550.5	452.7	559.4	515.7	506.9	487.4	538.0	442.7	581.3	547.4
	427.0			422.3	533.1	500.5	401.3	465.0	558.5	562.9	566.3	572.9	560.3	518.6	521.0	592.0	482.9	613.8	574.3
	546.0			466.6		527.5	401.3	509.1	563.9	574.7	630.6	586.5	586.2	637.4	559.4	599.8	484.5		
	546.0			498.3		638.7	463.8	570.7											
	568.8						463.8												
							475.0												
							475.0												
							475.3												
							512.5												
							523.5												
							535.8												
							641.8												
**N**	9.0	5.0	5.0	8.0	6.0	8.0	16.0	8.0	7.0	7.0	7.0	7.0	7.0	7.0	7.0	7.0	7.0	6.0	6.0
**MEAN**	415.7	511.8	449.0	407.5	453.3	451.3	451.0	450.9	446.8	480.1	471.6	542.0	471.7	499.5	482.2	506.8	419.1	500.5	495.3
**SD**	102.4	64.8	86.3	52.6	57.5	89.8	80.7	65.4	87.1	87.5	85.6	40.3	74.8	65.6	43.9	73.8	58.6	86.7	56.0
