# Supplementary material for: Thermal Preference Ranges Correlate with Stable Signals of Universal Stress Markers in Lake Baikal Endemic and Holarctic Amphipods
Source: PLoS One. 2016 Oct 5;11(10):e0164226. doi: 10.1371/journal.pone.0164226 (PMC5051968; doi:10.1371/journal.pone.0164226)
Supplement: S6 Table — (PDF) [file pone.0164226.s006.pdf]

S6 Table **Set of raw data of glutathione S-transferase activity (in nKat/ mg protein) in amphipod species during exposure to gradual temperature changes.**

Species: *E.verrucosus*
Total number of animals 110
Number of animals/analisys 1

**Temperature, °C**	**0.5**	**1**	**2**	**3**	**4**	**5**	**6**	**9**	**11**	**13**	**15**	**17**	**19**	**21**	**23**	**25**	**27**	**29**
**Raw data, nKat/mg prot**	2.4	3.7	2.9	3.3	4.2	3.3	4.2	4.5	4.2	2.2	4.2	4.1	4.3	3.2	2.5	2.8	3.9	3.6
	2.9	4.0	3.1	3.5	4.4	4.0	4.2	4.5	4.3	2.5	4.8	4.7	4.4	3.5	2.8	3.5	4.5	4.0
	3.1	4.1	3.5	4.0	4.6	4.4	4.5	5.0	4.4	3.2	5.1	4.9	4.4	4.4	3.1	3.9	4.7	4.2
	3.3	4.5	4.4	4.8	4.9	4.7	4.6	5.0	5.0	3.5	5.2	5.0	5.9	4.4	3.6	3.9	5.4	4.6
	3.5	4.6	5.0	5.7	5.2	5.3	4.9	5.2	5.5	3.8	5.8	5.0	6.0	4.6	3.8	4.1	5.6	4.9
	3.8			5.7	5.4	5.4	5.0	5.2	5.5	3.8		5.1	6.0	5.1				
	3.8						5.2	5.7				5.7						
							5.2	5.7										
							5.6											
							5.6											
							5.8											
							6.4											
**N**	7.0	5.0	5.0	6.0	6.0	6.0	12.0	8.0	6.0	6.0	5.0	7.0	6.0	6.0	5.0	5.0	5.0	5.0
**MEAN**	3.3	4.2	3.8	4.5	4.8	4.5	5.1	5.1	4.8	3.1	5.0	4.9	5.2	4.2	3.2	3.6	4.8	4.3
**SD**	0.5	0.3	0.9	1.1	0.5	0.8	0.7	0.5	0.6	0.7	0.6	0.5	0.9	0.7	0.5	0.5	0.7	0.5

Species: *O. flavus*
Total number of animals 192
Number of animals/analisys 3

**Temperature, °C**	**0.5**	**1**	**2**	**4**	**6**	**8**	**10**	**12**	**14**	**16**	**18**	**20**	**22**
**Raw data, nKat/mg prot**	3.6	3.4	3.7	3.4	5.0	3.2	4.1	3.7	3.2	6.6	7.0	5.4	4.2
	4.5	3.9	3.9	3.9	5.2	3.8	4.5	4.2	4.6	6.8	6.7	5.7	6.1
	4.6	4.3	4.0	4.1	5.5	3.9	4.6	4.2	4.8	6.8	6.6	6.1	7.5
	5.5	4.3	4.2	4.6	5.5	4.1	4.6	4.2	4.8	6.8	6.5	6.1	9.0
	6.0	4.5		4.8	6.2	4.6	5.9	4.6	5.5	7.2	5.3	6.9	
				4.9									
**N**	5.0	5.0	4.0	6.0	5.0	5.0	5.0	5.0	5.0	5.0	5.0	5.0	4.0
**MEAN**	4.8	4.1	3.9	4.3	5.5	3.9	4.7	4.2	4.6	6.8	6.4	6.1	6.7
**SD**	1.0	0.5	0.2	0.6	0.5	0.5	0.7	0.3	0.8	0.2	0.6	0.6	2.1

Species: *G. lacustris*
Total number of animals 695
Number of animals/analisys 5

**Temperature, °C**	**0.5**	**1**	**2**	**3**	**4**	**5**	**6**	**9**	**11**	**13**	**15**	**17**	**19**	**21**	**23**	**25**	**27**	**29**	**31**
**Raw data, nKat/mg prot**	2.0	2.0	1.6	1.6	2.0	2.1	2.0	1.8	1.5	2.0	1.7	2.0	1.7	2.1	1.8	1.4	2.4	1.6	1.2
	2.1	2.4	2.2	2.1	2.1	2.5	2.0	1.8	1.8	2.0	1.9	2.7	1.8	2.3	2.4	1.6	2.5	1.9	1.4
	2.1	2.4	2.4	2.1	2.3	2.6	2.0	2.6	2.1	2.3	2.2	2.8	2.0	2.4	2.4	2.2	2.8	2.2	1.5
	2.2	2.6	3.0	2.1	2.6	2.9	2.1	2.7	2.2	2.5	2.7	2.8	2.2	2.5	2.5	2.4	3.2	2.6	1.6
	2.7	2.9	3.4	2.3	2.7	3.0	2.2	2.7	2.3	2.5	2.8	3.0	2.5	2.7	2.7	2.5	3.6	2.7	2.2
	2.7			2.7	3.2	3.0	2.4	2.9	2.9	3.2	3.2	3.3	2.8	2.7	2.8	2.9	3.7	2.8	
	3.5			3.0		3.4	2.4	2.9	3.4	3.2	3.7	3.9	3.0	2.9	3.4	3.0	3.7		
	3.5			3.3		3.5	2.4	3.1											
	3.6						2.4												
							2.5												
							2.6												
							2.6												
							2.8												
							2.8												
							2.9												
							3.4												
**N**	9.0	5.0	5.0	8.0	6.0	8.0	16.0	8.0	7.0	7.0	7.0	7.0	7.0	7.0	7.0	7.0	7.0	6.0	5.0
**MEAN**	2.7	2.5	2.5	2.4	2.5	2.9	2.5	2.6	2.3	2.5	2.6	2.9	2.3	2.5	2.6	2.3	3.1	2.3	1.6
**SD**	0.7	0.3	0.7	0.5	0.4	0.5	0.4	0.5	0.6	0.5	0.7	0.6	0.5	0.3	0.5	0.6	0.6	0.5	0.4
