# Supplementary material for: Thermal Preference Ranges Correlate with Stable Signals of Universal Stress Markers in Lake Baikal Endemic and Holarctic Amphipods
Source: PLoS One. 2016 Oct 5;11(10):e0164226. doi: 10.1371/journal.pone.0164226 (PMC5051968; doi:10.1371/journal.pone.0164226)
Supplement: S7 Table — (PDF) [file pone.0164226.s007.pdf]

S7 Table **Set of raw data of lactate content (in µMol/g wet mass) in amphipods species during exposure to gradual temperature changes.**

Species: *E.verrucosus*
Total number of animals 109
Number of animals/analisys 1

**Temperature, °C**	**0.5**	**1**	**2**	**3**	**4**	**5**	**6**	**9**	**11**	**13**	**15**	**17**	**19**	**21**	**23**	**25**	**27**	**29**
**Raw data, uM/g w w**	0.41	0.18	0.13	0.13	0.14	0.09	0.05	0.16	0.25	0.04	0.44	0.44	0.25	0.80	0.47	0.50	0.41	0.70
	0.44	0.21	0.14	0.15	0.17	0.10	0.08	0.17	0.26	0.10	0.49	0.47	0.37	0.82	0.48	0.68	0.43	0.80
	0.54	0.27	0.15	0.16	0.18	0.13	0.09	0.20	0.28	0.13	0.50	0.49	0.40	0.92	0.50	0.68	0.51	1.06
	0.62	0.30	0.18	0.21	0.20	0.15	0.09	0.21	0.28	0.15	0.67	0.54	0.48	0.99	0.57	0.81	0.64	1.28
	0.62	0.31	0.27	0.26	0.23	0.19	0.12	0.28	0.36	0.17	0.67	0.58	0.55	1.01	0.71	0.99	0.68	1.37
							0.12	0.30	0.47	0.25	0.79	0.69	0.62	1.56	0.85	1.06	0.68	1.65
							0.16											
							0.17											
							0.17											
							0.19											
							0.19											
							0.20											
							0.25											
**N**	5.00	5.00	5.00	5.00	5.00	5.00	13.00	6.00	6.00	6.00	6.00	6.00	6.00	6.00	6.00	6.00	6.00	6.00
**MEAN**	0.53	0.25	0.17	0.18	0.19	0.13	0.15	0.22	0.32	0.14	0.60	0.53	0.45	1.02	0.60	0.79	0.55	1.14
**SD**	0.10	0.06	0.06	0.05	0.03	0.04	0.06	0.06	0.08	0.07	0.14	0.09	0.13	0.28	0.15	0.21	0.12	0.36

Species: *O. flavus*
Total number of animals 129
Number of animals/analisys 3

**Temperature, °C**	**0.5**	**1**	**2**	**4**	**6**	**8**	**10**	**12**	**14**	**16**	**18**	**20**	**22**
**Raw data, uM/g w w**	0.09	0.09	0.09	0.04	0.06	0.04	0.04	0.05	0.10	0.11	0.12	0.09	0.12
	0.11	0.11	0.12	0.06	0.06	0.05	0.04	0.05	0.12	0.12	0.17	0.13	0.22
	0.14	0.12	0.12	0.07	0.07	0.06	0.05	0.06	0.16	0.14	0.17	0.14	0.27
	0.17	0.14	0.14	0.07	0.08	0.07	0.05	0.06	0.16	0.18	0.18	0.15	0.29
	0.18	0.16	0.15	0.07	0.08	0.07	0.06	0.07	0.17	0.18	0.22	0.18	0.35
	0.29	0.21		0.08									
		0.23		0.08									
				0.08									
				0.09									
				0.10									
				0.11									
**N**	6.00	7.00	5.00	11.00	5.00	5.00	5.00	5.00	5.00	5.00	5.00	5.00	5.00
**MEAN**	0.16	0.15	0.12	0.08	0.07	0.06	0.05	0.06	0.14	0.15	0.17	0.14	0.25
**SD**	0.07	0.05	0.02	0.02	0.01	0.01	0.01	0.01	0.03	0.04	0.03	0.03	0.09

Species: *G. lacustris*
Total number of animals 665
Number of animals/analisys 5

**Temperature, °C**	**0.5**	**1**	**2**	**3**	**4**	**5**	**6**	**9**	**11**	**13**	**15**	**17**	**19**	**21**	**23**	**25**	**27**	**29**	**31**
**Raw data, uM/g w w**	0.02	0.04	0.01	0.04	0.07	0.09	0.07	0.13	0.07	0.13	0.07	0.29	0.08	0.26	0.16	0.27	0.10	1.70	2.80
	0.03	0.04	0.02	0.07	0.09	0.10	0.08	0.17	0.07	0.17	0.08	0.36	0.26	0.28	0.27	0.37	0.13	2.00	3.90
	0.05	0.05	0.03	0.08	0.12	0.12	0.09	0.19	0.08	0.21	0.14	0.44	0.32	0.28	0.32	0.42	0.24	2.60	4.06
	0.05	0.05	0.03	0.11	0.13	0.15	0.09	0.28	0.12	0.21	0.22	0.56	0.33	0.40	0.33	0.44	0.36	2.85	4.12
	0.05	0.06	0.05	0.12	0.14	0.18	0.11	0.31	0.12	0.22	0.28	0.61	0.36	0.40	0.37	0.48	0.62	2.87	4.76
	0.07	0.07	0.05	0.20	0.20	0.21	0.11	0.33	0.13	0.26	0.39	0.82	0.40	0.40	0.48	0.53	0.69	3.60	
				0.21			0.13	0.34	0.23	0.35	0.47	0.83	0.48	0.46					
							0.18					0.96		0.58					
							0.22												
							0.25												
							0.26												
							0.33												
							0.34												
							0.37												
							0.40												
							0.42												
**N**	6.00	6.00	6.00	7.00	6.00	6.00	16.00	7.00	7.00	7.00	7.00	8.00	7.00	8.00	6.00	6.00	6.00	6.00	5.00
**MEAN**	0.05	0.05	0.03	0.12	0.12	0.14	0.21	0.25	0.12	0.22	0.23	0.61	0.32	0.38	0.32	0.42	0.36	2.60	3.93
**SD**	0.02	0.01	0.02	0.07	0.05	0.05	0.13	0.08	0.05	0.07	0.15	0.24	0.13	0.11	0.11	0.09	0.25	0.68	0.71
