# Supplementary material for: Thermal Preference Ranges Correlate with Stable Signals of Universal Stress Markers in Lake Baikal Endemic and Holarctic Amphipods
Source: PLoS One. 2016 Oct 5;11(10):e0164226. doi: 10.1371/journal.pone.0164226 (PMC5051968; doi:10.1371/journal.pone.0164226)
Supplement: S8 Table — (PDF) [file pone.0164226.s008.pdf]

S8 Table **Set of raw data of lactate dehydrogenase activity (in nKat/ mg protein) in amphipods species during exposure to gradual temperature changes.**

Species: *E.verrucosus*
Total number of animals 119
Number of animals/analisys 1

Temperature, °C	0.5	1	2	3	4	5	6	9	11	13	15	17	19	21	23	25	27	29
Raw data, nKat/mg prot	221.8	356.1	642.7	617.4	300.1	324.1	212.7	252.7	278.8	158.7	113.8	110.2	138.9	126.9	85.2	136.1	174.3	145.3
	257.4	362.6	770.9	667.0	480.0	398.2	307.9	317.5	322.9	166.7	228.5	158.4	141.1	138.1	117.0	139.6	187.6	184.0
	371.6	370.8	832.8	792.7	516.8	424.2	316.7	318.5	328.4	168.7	236.0	180.8	142.6	172.0	166.0	176.0	196.2	190.0
	449.3	490.1	878.5	802.4	558.0	428.0	333.7	324.0	367.7	177.3	238.0	213.0	166.0	175.5	168.1	178.3	205.0	196.4
	478.4	509.8	878.5	871.8	649.2	486.2	367.3	326.0	378.6	215.6	239.5	277.8	180.1	192.4	181.1	182.3	212.2	227.1
	507.8	541.6	890.1	959.8	842.9	512.0	405.4	327.1	387.0	226.1	275.0	342.5	224.9	227.7	272.1	190.8	256.5	
	557.8						416.0	415.1			327.6							
	705.9						425.9											
	735.5						426.9											
							482.8											
							494.2											
							508.4											
							556.0											
N	9.0	6.0	6.0	6.0	6.0	6.0	13.0	7.0	6.0	6.0	7.0	6.0	6.0	6.0	6.0	6.0	6.0	5.0
MEAN	476.2	438.5	815.6	785.2	557.8	428.8	404.1	325.8	343.9	185.5	236.9	213.8	165.6	172.1	164.9	167.2	205.3	188.6
SD	167.3	76.9	87.2	115.8	165.2	60.8	91.7	43.8	37.7	25.7	59.6	77.0	30.5	33.5	58.3	21.3	25.9	26.3

Species: *O. flavus*
Total number of animals 192
Number of animals/analisys 3

Temperature, °C	0.5	1	2	4	6	8	10	12	14	16	18	20	22
Raw data, nKat/mg prot	212.7	290.8	352.3	126.6	169.6	215.6	552.4	270.4	123.9	178.3	127.7	80.7	41.2
	250.0	390.0	503.1	212.5	199.3	270.0	550.0	366.9	215.0	200.0	167.9	125.0	52.7
	300.0	474.7	505.0	222.0	230.0	270.0	650.0	370.0	231.0	212.7	210.0	130.0	55.9
	331.6	477.0	669.2	243.8	240.5	293.0	680.0	385.2	246.7	213.5	230.0	141.8	76.2
	469.6	688.6		275.6	332.9	307.6	832.7	464.3	290.3	227.3	394.3	181.3	
				296.0									
N	5.0	5.0	4.0	6.0	5.0	5.0	5.0	5.0	5.0	5.0	5.0	5.0	4.0
MEAN	312.8	464.2	507.4	229.4	234.5	271.2	653.0	371.4	221.4	206.4	226.0	131.8	56.5
SD	88.4	131.3	112.1	54.3	55.1	31.3	103.7	61.7	54.8	16.5	91.3	32.3	12.6

Species: *G. lacustris*
Total number of animals 675
Number of animals/analisys 5

Temperature, °C	0.5	1	2	3	4	5	6	9	11	13	15	17	19	21	23	25	27	29	31
Raw data, nKat/mg prot	173.9	191.7	279.6	206.9	177.8	200.7	188.7	255.6	162.7	200.6	291.8	124.2	53.0	117.2	225.8	119.7	82.7	54.4	44.2
	187.4	236.6	299.1	208.6	238.2	262.3	253.5	279.4	201.2	204.1	303.7	148.3	87.6	129.1	251.2	244.9	112.6	59.9	65.0
	227.0	250.7	304.6	212.8	260.2	275.2	272.5	299.8	300.1	233.1	325.0	161.0	89.2	132.8	265.7	268.5	124.8	86.0	100.2
	267.5	269.9	317.1	213.8	266.4	294.5	296.3	397.2	304.9	301.0	329.8	162.6	111.0	162.4	285.0	335.6	144.7	123.1	105.0
	324.7	312.6	324.6	218.9	276.4	316.7	323.6	426.5	357.1	302.1	334.3	181.8	150.5	187.6	315.0	342.6	167.9	126.1	137.4
	331.6	383.6	335.2	259.7	306.5	350.8	324.9	468.8	391.1	372.9	354.7	190.9	170.9	242.7	318.1	387.1	181.3	135.4	165.0
	328.5		344.1	288.1	355.7	384.1	368.1	473.5	417.7	400.0					351.8	388.3	181.4	142.5	
				362.7		399.2	385.3			413.0						447.7	226.6	179.0	
							412.3												
N	7.0	6.0	7.0	8.0	7.0	8.0	9.0	7.0	7.0	8.0	6.0	6.0	6.0	6.0	7.0	8.0	8.0	8.0	6.0
MEAN	262.9	274.2	314.9	246.4	268.7	310.4	313.9	371.5	305.0	303.4	323.2	161.5	110.4	162.0	287.5	316.8	152.8	113.3	102.8
SD	63.0	60.9	20.6	51.7	51.2	62.1	66.1	85.0	87.7	80.3	20.5	21.8	39.9	43.0	40.4	96.5	42.8	40.3	40.7
