# Supplementary material for: Thermal Preference Ranges Correlate with Stable Signals of Universal Stress Markers in Lake Baikal Endemic and Holarctic Amphipods
Source: PLoS One. 2016 Oct 5;11(10):e0164226. doi: 10.1371/journal.pone.0164226 (PMC5051968; doi:10.1371/journal.pone.0164226)
Supplement: S9 Table — (PDF) [file pone.0164226.s009.pdf]

S9 Table **Correlation analisys**

Species *E. verrucosus*

Type of experiment gradual hypothermia

	Hsp70	Lactate	LDH	Peroxidase	Catalase	Temperature
Hsp70		-0,89	-0,38	0,94	1,00	0,99
Lactate	-0,89		-0,08	-0,68	-0,88	-0,94
LDH	-0,38	-0,08		0,41	-0,50	-0,27
Peroxidase	0,94	-0,68	0,41		0,94	0,89
Catalase	1,00	-0,88	-0,50	0,94		0,99
Temperature	0,99	-0,94	-0,27	0,89	0,99	

Type of experiment gradual hyperthermia

	Hsp70	Lactate	LDH	Peroxidase	Catalase	Temperature
Hsp70		-0,66	0,55	-0,16	0,33	-0,62
Lactate	-0,66		-0,85	0,65	0,22	0,98
LDH	0,55	-0,85		-0,72	-0,50	-0,89
Peroxidase	-0,16	0,65	-0,72		0,33	0,72
Catalase	0,33	0,22	-0,50	0,33		0,22
Temperature	-0,62	0,98	-0,89	0,72	0,22	

Species *O. flavus*

Type of experiment gradual hypothermia

	Hsp70	Lactate	LDH	Peroxidase	Catalase	Temperature
Hsp70		0,96	0,01	0,42	-0,78	-0,93
Lactate	0,96		0,29	0,65	-0,92	-1,00
LDH	0,01	0,29		0,91	-0,64	-0,37
Peroxidase	0,42	0,65	0,91		-0,89	-0,71
Catalase	-0,78	-0,92	-0,64	-0,89		0,95
Temperature	-0,93	-1,00	-0,37	-0,71	0,95	

Type of experiment gradual hyperthermia

	Hsp70	Lactate	LDH	Peroxidase	Catalase	Temperature
Hsp70		0,69	-0,08	1,00	0,96	0,69
Lactate	0,69		-0,77	0,69	0,86	0,77
LDH	-0,08	-0,77		-0,08	-0,35	-0,47
Peroxidase	1,00	0,69	-0,08		0,96	0,69
Catalase	0,96	0,86	-0,35	0,96		0,74
Temperature	0,69	0,77	-0,47	0,69	0,74	

Species *G. lacustris*

Type of experiment gradual hypothermia

	Hsp70	Lactate	LDH	Peroxidase	Catalase	Temperature
Hsp70		0,27	0,62	0,18	0,31	0,61
Lactate	0,27		-0,58	1,00	1,00	0,93
LDH	0,62	-0,58		-0,65	-0,55	-0,24
Peroxidase	0,18	1,00	-0,65		0,99	0,89
Catalase	0,31	1,00	-0,55	0,99		0,94
Temperature	0,61	0,93	-0,24	0,89	0,94	

Type of experiment gradual hyperthermia

	Hsp70	Lactate	LDH	Peroxidase	Catalase	Temperature
Hsp70		0,93	-0,73	-0,21	0,47	0,72
Lactate	0,93		-0,74	-0,34	0,51	0,72
LDH	-0,73	-0,74		0,62	-0,80	-0,91
Peroxidase	-0,21	-0,34	0,62		-0,22	-0,39
Catalase	0,47	0,51	-0,80	-0,22		0,91
Temperature	0,72	0,72	-0,91	-0,39	0,91	

***

r values were visualised with blue columns in case of positive correlation and red columns in case of negative correlation
